# Hypothalamic ER–associated degradation regulates POMC maturation, feeding, and age-associated obesity

**DOI:** 10.1172/JCI96420

**Published:** 2018-02-19

**Authors:** Geun Hyang Kim, Guojun Shi, Diane R.M. Somlo, Leena Haataja, Soobin Song, Qiaoming Long, Eduardo A. Nillni, Malcolm J. Low, Peter Arvan, Martin G. Myers, Ling Qi

**Affiliations:** 1 Department of Molecular & Integrative Physiology, University of Michigan Medical School, Ann Arbor, Michigan, USA.; 2 Division of Nutritional Sciences, Cornell University, Ithaca, New York, USA.; 3 Division of Metabolism, Endocrinology & Diabetes, Department of Internal Medicine, University of Michigan Medical School, Ann Arbor, Michigan, USA.; 4 Cam-Su Genomic Resource Center, Soochow University, Suzhou, Jiangsu, China.; 5 The Warren Alpert Medical School, Department of Medicine, Molecular Biology, Cell Biology and Biochemistry, Brown University, Providence, Rhode Island, USA.

**Keywords:** Cell Biology, Metabolism, Monogenic diseases, Mouse models, Protein misfolding

## Abstract

Pro-opiomelanocortin (POMC) neurons function as key regulators of metabolism and physiology by releasing prohormone-derived neuropeptides with distinct biological activities. However, our understanding of early events in prohormone maturation in the ER remains incomplete. Highlighting the significance of this gap in knowledge, a single POMC cysteine-to-phenylalanine mutation at position 28 (POMC-C28F) is defective for ER processing and causes early onset obesity in a dominant-negative manner in humans through an unclear mechanism. Here, we report a pathologically important role of Sel1L-Hrd1, the protein complex of ER-associated degradation (ERAD), within POMC neurons. Mice with POMC neuron–specific Sel1L deficiency developed age-associated obesity due, at least in part, to the ER retention of POMC that led to hyperphagia. The Sel1L-Hrd1 complex targets a fraction of nascent POMC molecules for ubiquitination and proteasomal degradation, preventing accumulation of misfolded and aggregated POMC, thereby ensuring that another fraction of POMC can undergo normal posttranslational processing and trafficking for secretion. Moreover, we found that the disease-associated POMC-C28F mutant evades ERAD and becomes aggregated due to the presence of a highly reactive unpaired cysteine thiol at position 50. Thus, this study not only identifies ERAD as an important mechanism regulating POMC maturation within the ER, but also provides insights into the pathogenesis of monogenic obesity associated with defective prohormone folding.

## Introduction

Hypothalamic pro-opiomelanocortin (POMC) neurons critically mediate leptin signaling and regulate feeding behavior and systemic metabolic homeostasis via the secretion of several bioactive neuropeptides derived from the POMC protein precursor (1–4). POMC-derived neuropeptides, including adrenocorticotropic hormone (ACTH), α/β melanocyte–stimulating hormone (α/β-MSH), and β-endorphin (5, 6), are critical for energy homeostasis, and both POMC-deficient rodent models and human patients with POMC mutations invariably exhibit hyperphagia and marked obesity (4, 7–15). Some of these mutations would be expected to alter POMC folding and turnover in the ER; for example, an autosomal dominant POMC cysteine-to-phenylalanine mutation at position 28 (POMC-C28F) has been identified in humans with early onset obesity (16). Furthermore, diet-induced obesity is also associated with an accumulation of POMC in the ER of POMC neurons (17, 18). However, while the post-Golgi endoproteolytic cleavage of POMC has been well studied, the pathway for conformational maturation of POMC within the ER remains unmapped, and the molecular factors that regulate POMC export from the ER remain unclear.

Quality control systems normally maintain homeostasis and proteostasis in the ER over the lifetime of an organism. Disruption of ER homeostasis is a hallmark of several pathologies, including metabolic disorders and neurodegenerative diseases (19). Quality control systems in the ER consist mainly of the unfolded protein response (UPR) and ER-associated degradation (ERAD). UPR detects misfolded proteins in the ER and coordinates responses to environmental cues and stresses differently depending on cell and tissue type (19). Specifically, in the hypothalamic region of the brain, elevated UPR activity has been shown to potentiate diet-induced obesity (17, 20–23) and mice overexpressing UPR effector X-box binding protein 1 (XBP1) in POMC neurons are resistant to diet-induced obesity via cell- and non–cell-autonomous mechanisms (24). When the UPR sensor Ire1α is deleted in POMC neurons, mice are susceptible to diet-induced obesity, with reduced energy expenditure (25). However, another group reported that POMC neuron-specific *Ire1a*-KO mice are resistant to diet-induced obesity with increased energy expenditure (26). The basis for these contradictory outcomes is unclear, but the presence of such controversy highlights the need to clarify the physiological role or roles of ER quality control systems in POMC neurons.

The other major ER quality control mechanism utilizes the ERAD system comprising the E3 ligase hydroxymethylglutaryl reductase degradation protein 1 (Hrd1) and its cofactor suppressor-enhancer of lin-like 1 (Sel1L) protein. This complex (Sel1L-Hrd1) represents the most conserved branch of ERAD, which recognizes and targets ER proteins for cytosolic proteasomal degradation via the ubiquitin-proteasome system (27, 28). The cofactor Sel1L, a single-spanning ER transmembrane protein, is required for the stability of Hrd1 (29). While the biochemical processes of ERAD have been extensively investigated over the past 2 decades, we are only just beginning to appreciate the physiological role of ERAD in vivo (28). Germline loss of Sel1L or Hrd1 is embryonic lethal (30, 31) and causes premature death when induced in adult mouse models (29, 32). Subsequent characterization of cell type–specific Sel1L- (29, 33–36) and Hrd1-KO mouse models (32, 37, 38) has revealed cell type–specific significance of Sel1L-Hrd1 ERAD in health and disease (28). For example, mice with Sel1L deficiency in adipocytes exhibit lipodystrophy and hyperlipidemia due to the ER retention of lipoprotein lipase (LPL) (33). Further, mice with Sel1L ablation in arginine-vasopressin (AVP) neurons progressively develop polyuria and polydipsia — characteristics of diabetes insipidus, due to a maturation defect of AVP precursor, proAVP in the ER (39). These studies not only point to the (patho-)physiological importance of cell type–specific ERAD in health and disease (28), but also highlight our limited understanding of the role of ERAD in physiology.

In this study, we report a role of Sel1L-Hrd1 ERAD in POMC neurons that is tightly linked to the mechanism underlying the conformational maturation of POMC within the ER. POMC neuron–specific Sel1L-KO (*Sel1L^fl/fl^ POMC*, hereafter referred to as *Sel1L^POMC^*) mice develop hyperphagia and age-associated obesity even on a low-fat diet (LFD), with intracellular retention of POMC. Moreover, biochemical studies demonstrate that POMC is a bona fide ERAD substrate and that disease-associated POMC-C28F evades ERAD quality control and forms extensive intermolecular disulfide bond-mediated high molecular-weight aggregates via the unpaired free cysteine at residue 50 (C50). This study points to a previously unappreciated role of ERAD in POMC neurons to influence systemic energy balance via endogenous prohormone maturation.

## Results

### Sel1L-Hrd1 ERAD expression in POMC neurons is responsive to physiological feeding signals.

**Figure 1:**
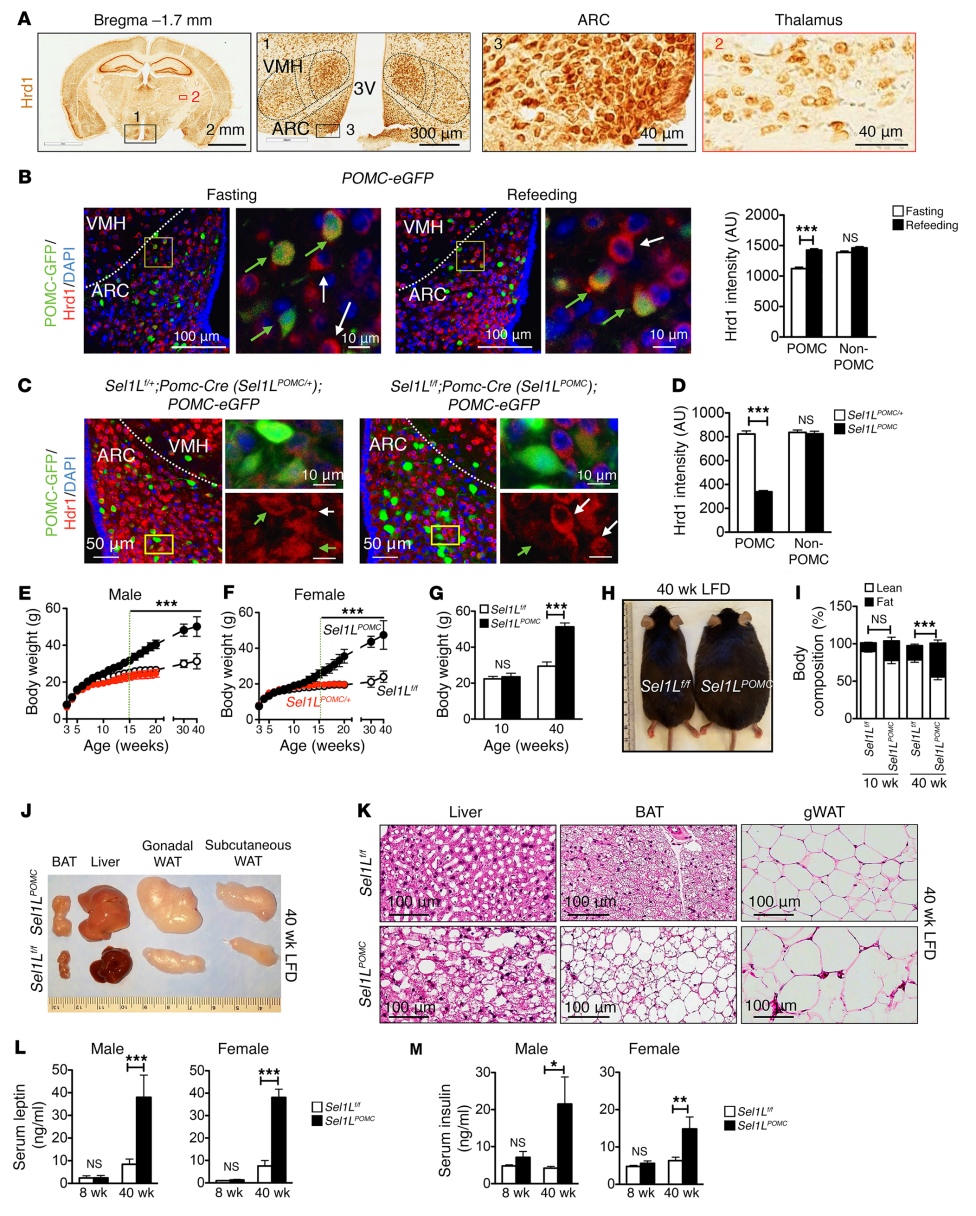
POMC neuron–specific *Sel1L^POMC^* mice develop age-associated obesity and hyperleptinemia. (**A**) Representative images of immunohistochemical staining of Hrd1 in the brains of 7-week-old C57BL/6J mice on LFD (*n* = 2 each group). Zoomed-in images of ARC and thalamus are shown on the right. 3V, third ventricle. Representative images of negative control IgG are shown in Supplemental Figure 1A. (**B**) Representative images of Hrd1 staining in the ARC of 7-week-old *POMC-eGFP* reporter mice after an overnight fast with or without 6-hour refeeding. Quantitation of Hrd1 signals in POMC neurons (green arrows) and non-POMC neurons (white arrows) shown on the right (*n* = 2 mice each group, 70 neurons each mouse). (**C**) Representative images of Hrd1 staining in the ARC of 8-week-old *Sel1L^POMC^;POMC-eGFP* and control *Sel1L^POMC/+^*;*POMC-eGFP* mice on LFD (*n* = 3–4 each group). Green arrows point to POMC neurons; white arrows point to non-POMC neurons. (**D**) Quantitation of Hrd1 level shown in **C** in POMC and non-POMC neurons in the ARC (*n* = 70 and *n* = 100 neurons per mouse, *n* = 3–4 mice each). (**E** and **F**) Growth curve of *Sel1L^fl/fl^* (*n* = 5), heterozygous *Sel1L^POMC/+^* (*Sel1L^fl/+^;Pomc-Cre*, *n* = 3), and *Sel1L^POMC^* mice (*n* = 7) on LFD. In **E**, a green dotted line marks the age at which *Sel1L^POMC^* mice became significantly more obese. (**G**) Body weight of 10- and 40-week-old mice on LFD. (**H**) Representative image of 40-week-old mice on LFD. (**I**) Body composition of 10-week-old (*n* = 3 each) and 40-week-old (*n* = 6–7 each) male mice on LFD. (**J** and **K**) Representative images of peripheral tissues (**J**) and H&E images of peripheral tissues (**K**) from 40-week-old mice (*n* = 3 each group). gWAT, gonadal WAT. (**L** and **M**) Serum leptin (**L**) and insulin (**M**) levels of 8- and 40-week-old mice of both sexes fed ad libitum LFD (*n* = approximately 4–6 each group). Values are shown as mean ± SEM. *P < 0.05; **P < 0.01; ***P < 0.001, 2-way ANOVA.

Hrd1 is ubiquitously expressed in the cytoplasm of neuronal cells throughout the brain, but is highly enriched in the hypothalamic arcuate nucleus (ARC) and ventromedial hypothalamus (VMH), both of which are critical regions for feeding behavior (40) (Figure 1A, with negative control staining in Supplemental Figure 1A). POMC neurons are predominantly located in the ARC of the hypothalamus and are key mediators of the leptin effect in vivo. A negative control with irrelevant antibody IgG is shown in Supplemental Figure 1A. Feeding induced mRNA levels of both *Pomc* and *Sel1L* in the ARC, but not of the ER chaperone *BiP* (Supplemental Figure 1B). Using *POMC-eGFP* reporter mice with GFP-labeled POMC neurons (41), we also found that Hrd1 protein levels were significantly increased in POMC neurons in the ARC in response to refeeding following an overnight fast, but were not increased in non-POMC neurons (Figure 1B). Moreover, injection of leptin induced *Pomc* gene expression after 6 hours, as expected, but also induced Sel1L-Hrd1 ERAD gene expression (Supplemental Figure 1C). These data demonstrate that Sel1L-Hrd1 ERAD is expressed in POMC neurons and is responsive to physiological cues.

### Sel1L^POMC^ mice develop age-associated obesity and hyperleptinemia.

To delineate the role of ERAD in POMC neurons, we generated *Sel1L^POMC^* mice by crossing *Sel1L^fl/fl^* mice with the *Pomc-Cre* driver line, with Cre-negative *Sel1L^fl/fl^* or heterozygous *Sel1L^POMC/+^* littermates as controls. Additionally, we crossed *Sel1L^POMC^* mice with the *POMC-eGFP* reporter mice to generate *Sel1L^POMC^* or control heterozygous *Sel1L^POMC/+^* mice with GFP-labeled POMC neurons. Consistent with previous findings on the indispensable role of Sel1L in Hrd1 stability (29, 33–35), deletion of Sel1L significantly reduced the levels of Hrd1 by over 60% specifically in the POMC neurons (Figure 1, C and D).

On LFD (13% calories from fat) ad libitum, both sexes of *Sel1L^fl/fl^* and *Sel1L^POMC^* mice had comparable weight for the first 3 months of age (Figure 1, E and F). However, while gross appearance was similar, histology showed that white adipose tissue (WAT) and brown adipose tissue (BAT) from *Sel1L^POMC^* mice had relatively larger lipid droplets by 10 weeks of age (Supplemental Figure 2A). Around 15 weeks of age, both male and female *Sel1L^POMC^* mice developed age-associated obesity (Figure 1, E and F). By 40 weeks of age, *Sel1L^POMC^* mice reached 50 g body weight compared with 25–30 g of *Sel1L^fl/fl^* littermates (Figure 1, G and H) and fat mass in *Sel1L^POMC^* mice had expanded to nearly 50% of body weight (Figure 1I and Supplemental Figure 2B). Tissues such as BAT, liver, and WAT were enlarged, with a large proportion of lipids in 40-week-old *Sel1L^POMC^* mice (Figure 1, J and K, and Supplemental Figure 2C). Of note, POMC neuron–specific Sel1L heterozygous (*Sel1L^POMC/+^*) mice exhibited body weight and body composition comparable to those of *Sel1L^fl/fl^* littermates (Figure 1, E and F, and Supplemental Figure 2B), suggesting that 1 copy of Sel1L is sufficient for ERAD function in POMC neurons.

At 8 to 12 weeks of age, nonobese *Sel1L^POMC^* mice were equally glucose tolerant and insulin sensitive compared with *Sel1L^fl/fl^* littermates (Supplemental Figure 3, A–C). However, at approximately 20–23 weeks of age, when *Sel1L^POMC^* mice became obese (Supplemental Figure 3A), they became insulin resistant, but remained glucose tolerant (Supplemental Figure 3, D and E). Serum leptin and insulin levels were comparable between the 2 cohorts at 8 weeks of age, but thereafter, *Sel1L^POMC^* mice developed hyperleptinemia and hyperinsulinemia (Figure 1, L and M). Thus, Sel1L deficiency in POMC neurons leads to age-associated adiposity accompanied by hyperleptinemia, hyperinsulinemia, and insulin resistance.

### Sel1L^POMC^ mice are hyperphagic, with impaired leptin response.

**Figure 2:**
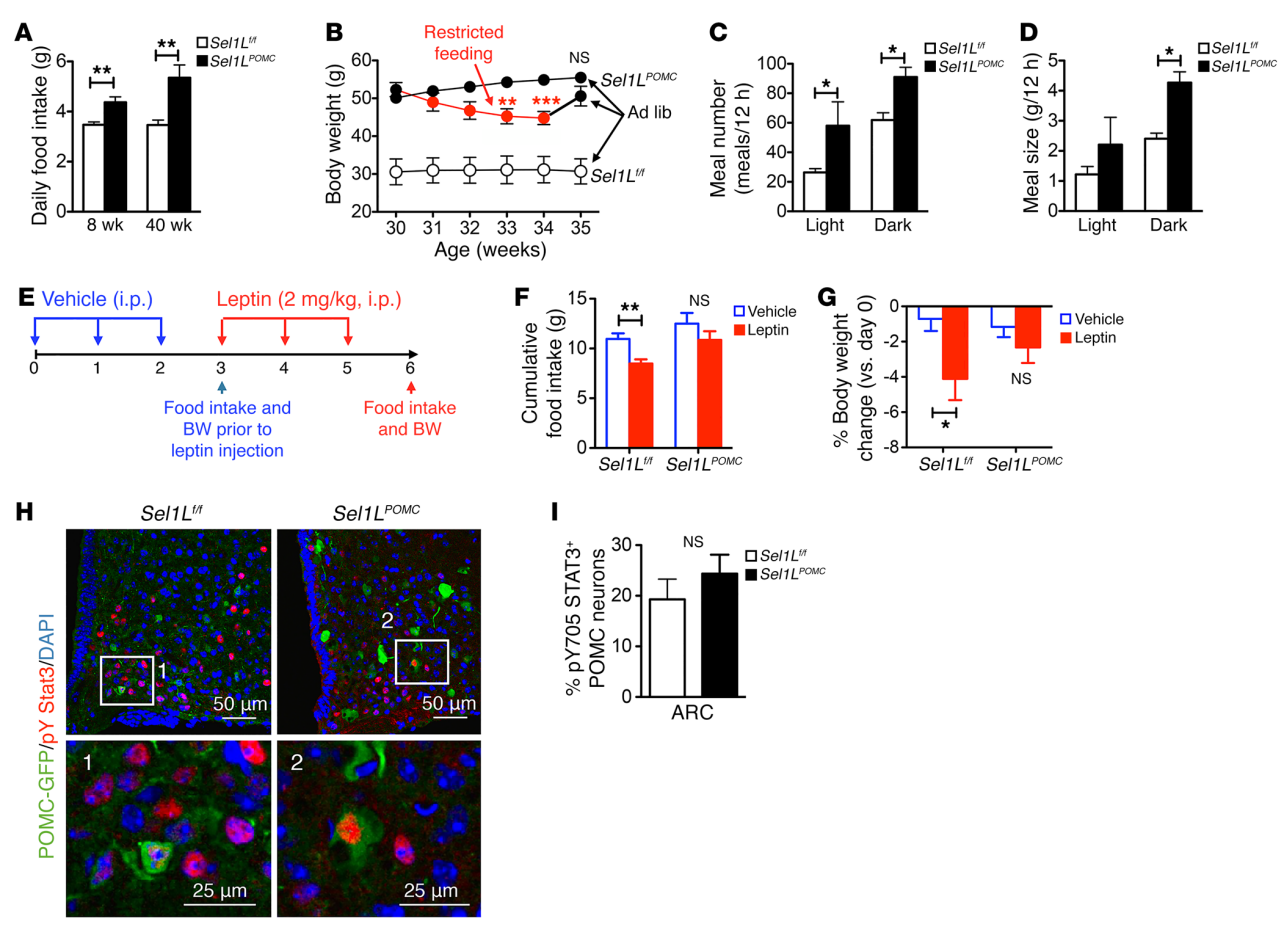
*Sel1L^POMC^* mice exhibit hyperphagia with impaired leptin sensitivity. (**A**) Daily food consumption of 8- and 40-week-old mice on LFD (*n* = approximately 5–6 each group). (**B**) Body weight of 30-week-old *Sel1L^POMC^* mice under food restricted for 4 weeks (red line) followed by 1-week ad libitum on LFD (black line) or ad libitum LFD for 5 weeks (black line) (*n* = 3 each group). *Sel1L^fl/fl^* mice fed ad libitum LFD were included as controls (white circles). For clarity, only statistical analyses comparing restricted feeding to ad libitum for *Sel1L^POMC^* mice are shown. (**C** and **D**) Meal number (**C**) and size (**D**) of 8-week-old mice on LFD (*n* = 3–4 each group) as measured using the CLAMS metabolic cages. (**E**–**I**) Leptin response analyses in which mice approximately 7 to 9 weeks old were i.p. injected daily with leptin (2 mg/kg body weight) for 3 days and then perfused with fixative 30 minutes after the last injection. (**E**) Schematic diagram for the experiment. BW, body weight. (**F**) Cumulative food intake and (**G**) percentage of body weight change following 3 daily vehicle (saline) and leptin injections of 8-week-old mice (*n* = approximately 6–8 each group). (**H** and **I**) Representative images of immunofluorescent staining of pY705 STAT3 in the ARC of *POMC-eGFP* mice with or without Sel1L (*n* = 2 each group). Quantitation of the percentage of pY705 STAT3-positive POMC neurons of total POMC neurons in the ARC are shown in **I**. Values are shown as mean ± SEM. *P < 0.05; **P < 0.01; ***P < 0.001, 2-way ANOVA (**C**, **D**, **F**, **G**) or Student’s *t* test (**A**, **B**, **I**).

We next investigated how Sel1L deficiency in POMC neurons leads to age-associated obesity, first by measuring food intake and metabolic rates of *Sel1L^POMC^* mice. Even at 8 weeks of age, when their body weights were similar to those of *Sel1L^fl/fl^* littermates, *Sel1L^POMC^* mice consumed significantly more food on a daily basis (Figure 2A). If *Sel1L^POMC^* mice were restricted to the same amount of food consumed by WT mice (i.e., restricted feeding), obese *Sel1L^POMC^* mice demonstrated a significant reduction in body weight (Figure 2B). When free access to food was reintroduced, *Sel1L^POMC^* mice quickly regained body weight to levels similar to those of *Sel1L^POMC^* mice fed ad libitum throughout the experiment (Figure 2B). These data suggest that overeating indeed was responsible for age-associated obesity in *Sel1L^POMC^* mice.

Daily food intake is a factor of both the size and frequency of meals. A normal diurnal rhythm of eating was observed in *Sel1L^fl/fl^* littermates at the age of 8 weeks (Figure 2C). Interestingly, loss of Sel1L in POMC neurons led to increased meal frequency during both day and night (Figure 2C). The total amount of food consumed in 12 hours was significantly higher in *Sel1L^POMC^* mice at night (Figure 2D). However, the metabolic rate of *Sel1L^POMC^* mice, as measured by O_2_ consumption and CO_2_ production (normalized to lean mass), was not different from that of their *Sel1L^fl/fl^* littermates at 8 weeks of age (Supplemental Figure 4A). Respiratory exchange ratio (RER) and physical activity were also comparable between the 2 cohorts at 8 weeks of age (Supplemental Figure 4B). These parameters remained unchanged between the 2 cohorts, even at 40 weeks of age (Supplemental Figure 4, C and D).

Hypothalamic POMC neurons mediate leptin’s effect on food intake and body weight in the paraventricular nucleus (PVN) of the hypothalamus (41–43). To provide direct evidence linking Sel1L in POMC neurons to leptin signaling, we tested the leptin response in cohorts of 8-week-old animals (Figure 2E). Daily injection of leptin for 3 days significantly reduced food intake and body weight in *Sel1L^fl/fl^* mice, but not in *Sel1L^POMC^* mice (Figure 2, F and G), indicating an impaired leptin response in *Sel1L^POMC^* mice. However, leptin induced tyrosine phosphorylation of the transcription factor STAT3 (pY705 STAT3) in a similar number of POMC neurons between the cohorts (Figure 2, H and I), suggesting that the immediate leptin downstream signaling pathway in POMC neurons may not be significantly affected by Sel1L deficiency. Taken together, these data demonstrate that Sel1L deficiency in POMC neurons leads to age-associated obesity due to overeating, partially due to impaired leptin response at a step downstream of pY-STAT3.

### Sel1L^POMC^ mice do not exhibit elevated inflammation or cell death in the ARC.

**Figure 3:**
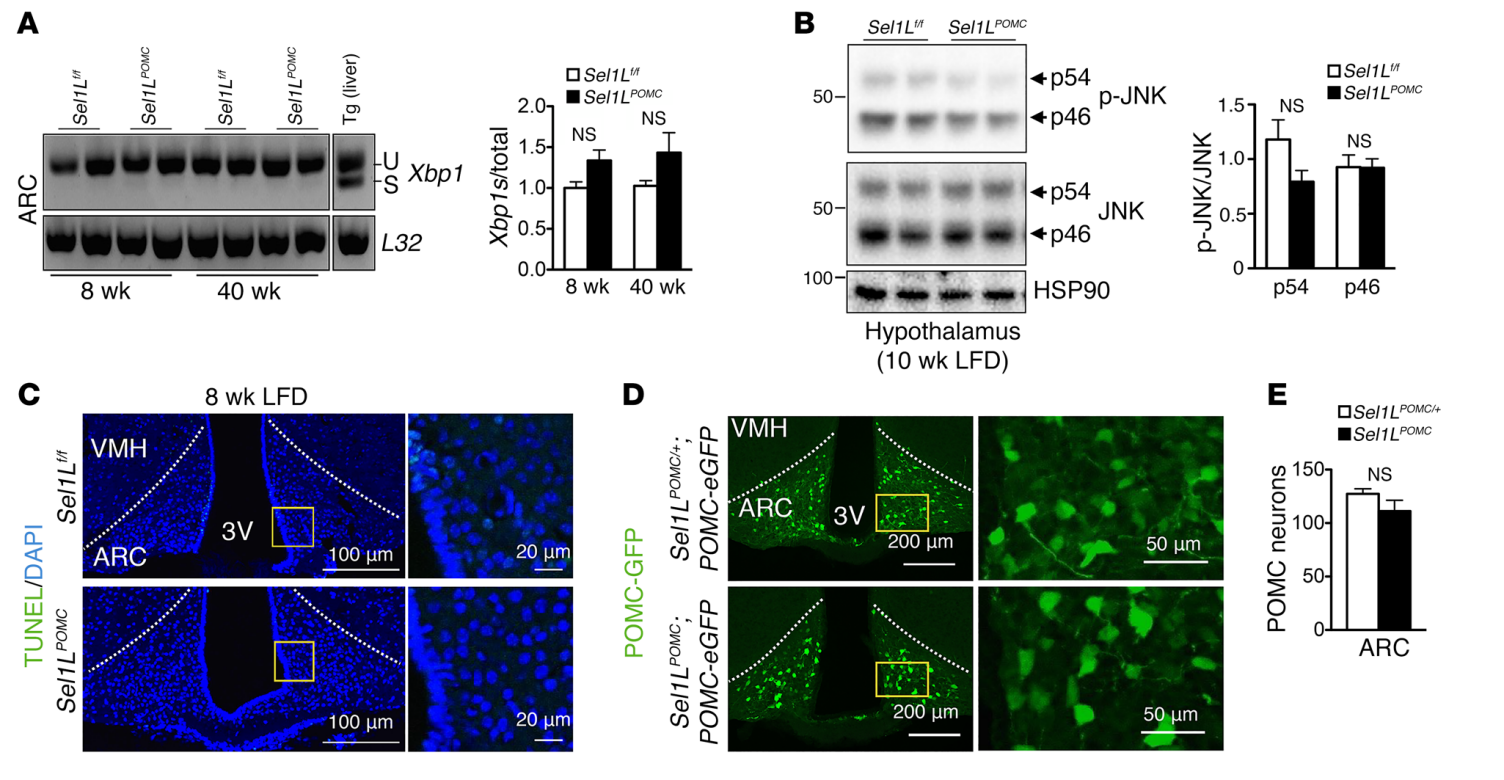
Sel1L deficiency in POMC neurons is not associated with elevated inflammation and cell death in ARC. (**A**) Reverse transcriptase PCR (RT-PCR) analysis of *Xbp1* mRNA splicing (u, unspliced; s, spliced) in ARC with quantitation shown on the right (*n* = 2–3 each group). Liver samples from mice injected with thapsigargin (Tg) were included as positive controls. (**B**) Western blot analysis of unphosphorylated and phosphorylated JNK (p-JNK) in the hypothalamus of 10-week-old mice on LFD with quantitation on the right (*n* = 3 each group, experiment was performed in duplicate). (**C**) Representative images of TUNEL analysis in the ARC of 8-week-old mice on LFD (*n* = approximately 4–5 each group). Quantitation of TUNEL-positive cells in ARC is shown in Supplemental Figure 5G. (**D**) Representative immunofluorescent images of GFP-positive POMC neurons in the ARC of 8-week-old *Sel1L^POMC^;POMC-eGFP* and control *Sel1L^POMC/+^;POMC-eGFP* mice on LFD with quantitation of the numbers of GFP-positive neurons in central ARC (bregma, –1.58/–1.94 mm) shown in **E** (*n* = 3–4 each group). Values are shown as mean ± SEM. Student’s *t* test was used.

As neuronal UPR and inflammation have been linked to diet-induced obesity in mice (44), we next asked whether similar associations may be present in our LFD cohorts. We measured *Xbp1* mRNA splicing in the ARC of mice at 8 weeks of age and found that there was a slight, but not significant, increase in *Xbp1* mRNA splicing in *Sel1L^POMC^* mice (Figure 3A). Phosphorylation of UPR sensor IRE1α and protein kinase RNA-like endoplasmic reticulum kinase (PERK) downstream effector eIF2α at 8 weeks was modestly increased in the ARC of *Sel1L^POMC^* mice compared with that in WT littermates (Supplemental Figure 5, A and B). Of note, IRE1α protein levels were increased in the absence of Sel1L, consistent with findings that IRE1α is an ERAD substrate (34, 35). Also at 8 weeks of age, BiP protein levels were doubled in the ARC of *Sel1L^POMC^* mice (Supplemental Figure 5, A and B). At 40 weeks of age, UPR activation was less evident (Figure 3A and Supplemental Figure 5, A and B). Collectively, these data demonstrate that Sel1L deficiency triggers a low level of ER stress in the ARC that appears adapted with age.

Next, we checked inflammatory status and cell death in the ARC. There was no difference in the phosphorylation of JNK (Figure 3B) as well as other inflammatory genes in the ARC of *Sel1L^POMC^* mice compared with WT littermates (Supplemental Figure 5, C and D), suggesting that the inflammatory tone of the ARC is not affected by Sel1L deficiency. Cell death, as measured by TUNEL staining and Western blot analysis of caspase 3 cleavage, was not detected in the ARC of *Sel1L^POMC^* mice at either 8 or 40 weeks of age (Figure 3C, Supplemental Figure 5, E and F; quantitation shown in Supplemental Figure 5, G and H). Using *Pomc-eGFP* reporter mice, we found that *Sel1L^POMC^* mice had a similar number of POMC neurons in the ARC (Figure 3, D and E). Hence, we conclude that the effect of Sel1L in POMC neurons on systemic energy metabolism is accompanied by a mild early ER stress response and is uncoupled from inflammation or loss of POMC neurons.

### Intracellular retention of POMC in the absence of Sel1L.

**Figure 4:**
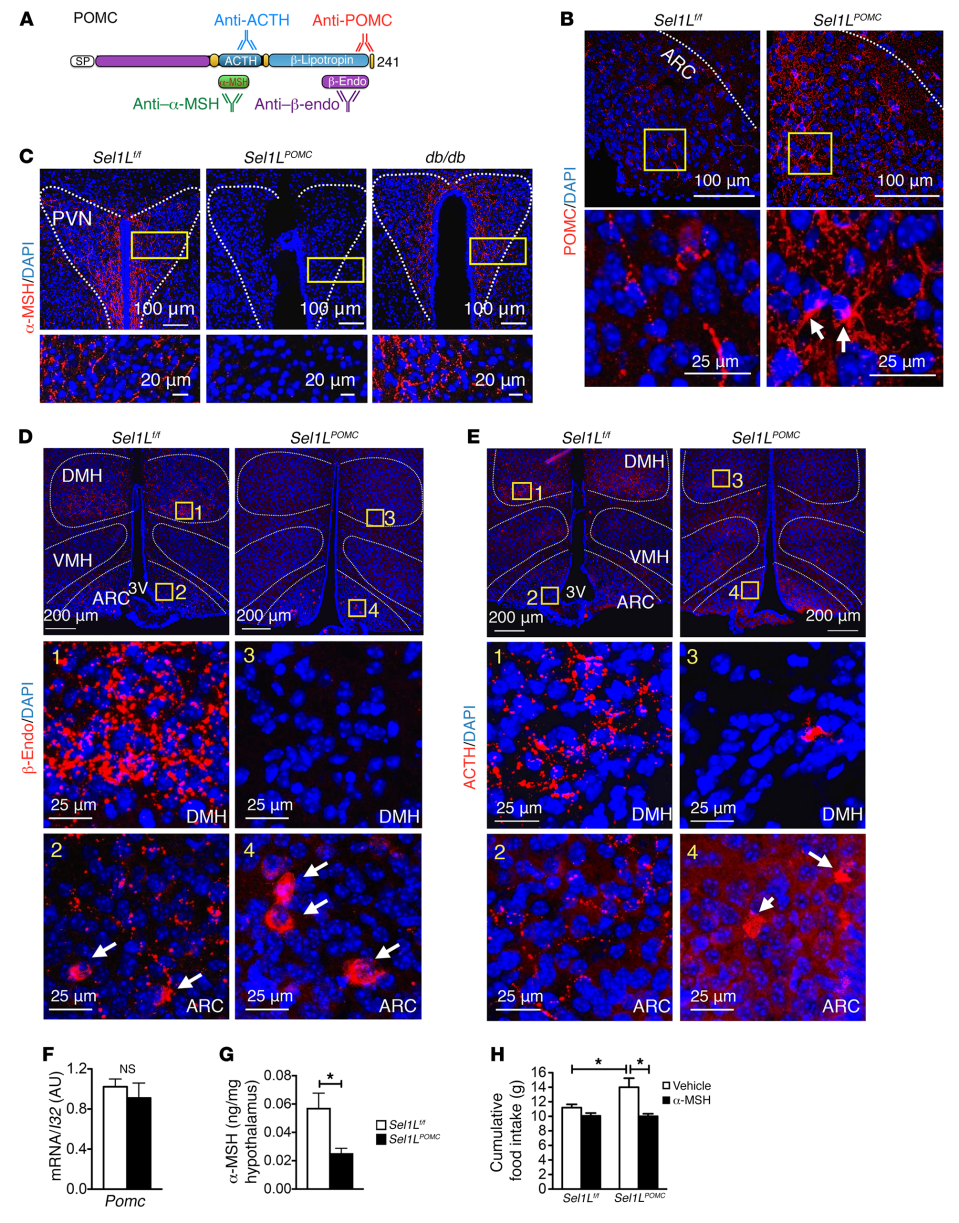
Intracellular retention of POMC in the absence of Sel1L. (**A**) Schematic diagram showing specific domains and processing derivatives of POMC recognized by various antibodies. SP, signal peptide; β-endo, β-endorphin. (**B**–**E**) Representative immunofluorescence images of (**B**) POMC as a prohormone (*n* = 5–6 each) in the ARC of mice approximately 5 to 10 weeks of age, (**C**) α-MSH in the axons of POMC neurons in the PVN of mice approximately 5 to 10 weeks of age (*n* = 4 each), (**D**) β-endorphin (*n* = 4–5 in each), and (**E**) ACTH (*n* = 2-3 each) in the ARC and DMH of mice approximately 5 to 8 weeks of age fed ad libitum LFD. White arrows indicate staining in the cell bodies of POMC neurons. Quantitation of POMC and neuropeptide signal intensity in cell bodies and axons are shown in Supplemental Figure 6, A–B. (**F**) *Pomc* mRNA level in the ARC of mice approximately 5 to 8 weeks of age fed ad libitum LFD (*n* = 4 each). (**G**) α-MSH levels in the hypothalamus of mice approximately 6 to 7 weeks of age, measured by ELISA (*n* = 3 each group). (**H**) Cumulative food intake in 8-week-old mice injected daily i.p. with α-MSH (1 mg/kg body weight) for 3 days (*n* = approximately 5–7 each group). Vehicle, saline. Values are shown as mean ± SEM. *P < 0.05, Student’s *t* test (**F** and **G**) or 2-way ANOVA (**H**).

Since neither inflammation nor cell death in the ARC was apparent in *Sel1L^POMC^* mice, we sought to explain the onset of obesity by other mechanisms. Given the role of POMC-derived neuropeptides in systemic energy homeostasis (6), we examined POMC maturation and trafficking using immunofluorescent staining for POMC (Figure 4A). Immunostaining using POMC-specific antibody revealed intracellular accumulation of POMC protein in *Sel1L^POMC^* neurons in the ARC (Figure 4B). Quantitation of POMC signal in neuronal cell body and axon in the ARC are shown in Supplemental Figure 6A. This was further confirmed using 2 additional POMC antibodies specific for neuropeptides β-endorphin and ACTH (panels 2 vs. 4 in Figure 4, D and E). In contrast, *Pomc* mRNA levels were unchanged or slightly reduced in the ARC of *Sel1L^POMC^* mice (Figure 4F), pointing to a defect in posttranslational POMC maturation in the absence of Sel1L.

The axons of POMC neurons project through the hypothalamus, especially the dorsomedial hypothalamus (DMH) and PVN (45). We next examined POMC processing using antibodies specific for POMC-derived endoproteolytic cleavage products: α-MSH, ACTH, and β-endorphin (Figure 4A). The immunostaining of α-MSH showed a punctate pattern, presumably in secretory granules in the axons of POMC neurons, in the PVN of WT mice (Figure 4C). In contrast, α-MSH was largely absent in the PVN of *Sel1L^POMC^* mice (Figure 4C). Importantly, the reduction of α-MSH staining in *Sel1L^POMC^* neurons was even more pronounced than that observed in leptin receptor–deficient *db/db* mice (Figure 4C), indicating that impaired leptin signaling alone cannot account for the dramatic reduction of POMC-derived peptides in *Sel1L^POMC^* axons. Similarly, staining of β-endorphin and ACTH in the ARC and DMH was evident and punctate in POMC-WT axons, but was markedly decreased in *Sel1L^POMC^* axons (panels 3 vs. 1, Figure 4, D and E). Quantitation of α-MSH, β-endorphin, and ACTH signal intensity in the axons is shown in Supplemental Figure 6B. Finally, in the absence of Sel1L, POMC (stained by the β-endorphin antibody) was accumulated in the ER, as demonstrated by the colocalization with ER chaperone XTP3-B (Supplemental Figure 6, C and D). The accumulation of XTP3-B protein levels in *Sel1L^POMC^* neurons was again consistent with a mild adaptive ER stress response to ERAD deficiency.

To further demonstrate the defect in POMC processing in *Sel1L^POMC^* neurons, we next performed the following 2 experiments. First, we measured the concentration of α-MSH in the hypothalamus using α-MSH–specific ELISA and found that α-MSH levels were indeed markedly reduced in *Sel1L^POMC^* mice (Figure 4G). Moreover, administration of recombinant α-MSH reversed hyperphagia of *Sel1L^POMC^* mice at 8 weeks of age (Figure 4H). Taken together, these data demonstrate that a primary defect caused by Sel1L deficiency in POMC neurons is a profound ER retention of POMC, resulting in a greatly diminished formation of mature, bioactive POMC-processing products.

### POMC is an endogenous substrate of Sel1L-Hrd1 ERAD.

**Figure 5:**
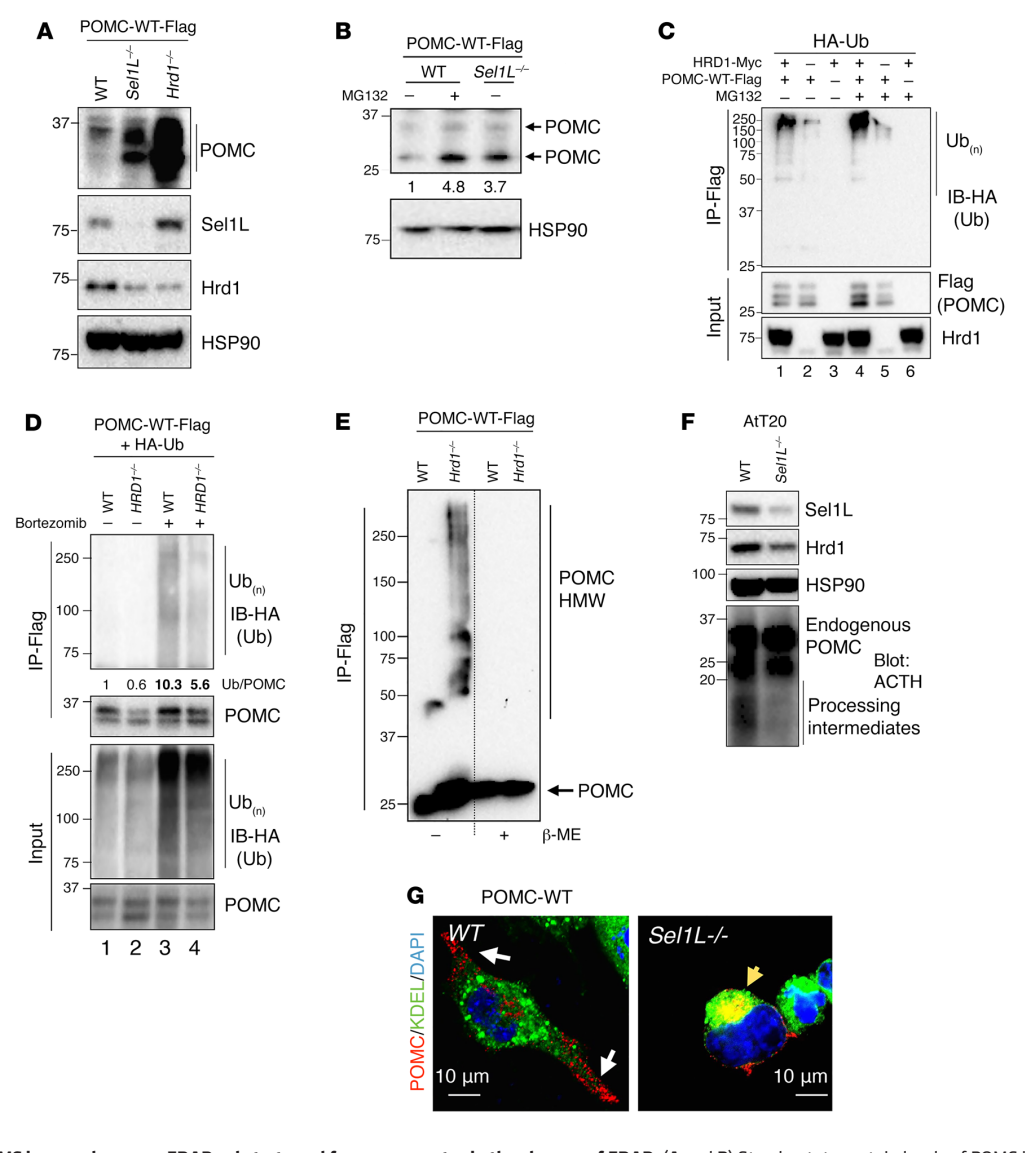
POMC is an endogenous ERAD substrate and forms aggregates in the absence of ERAD. (**A** and **B**) Steady-state protein levels of POMC in WT and ERAD-deficient N2a cells transfected with POMC-WT-Flag. In **B**, proteasomal inhibitor MG132 was added for the last 2 hours. mRNA levels of *Pomc* in **A** and **B** are shown in Supplemental Figure 6, E–F. (**C** and **D**) Ubiquitination of POMC by HRD1 in gain- (**C**) and loss-of-function (**D**) systems: Western blot of ubiquitin in POMC-Flag immunoprecipitates of HEK293T cells transfected with POMC-WT-Flag with or without HRD1. MG132 (**C**) or bortezomib (**D**) was added for the last 2 hours. In **D**, quantitation of the ratio of ubiquitination (Ub) signal intensity to POMC band intensity is shown in the lower panels. (**E**) Western blot analysis of POMC-Flag immunoprecipitates in transfected WT and *Hrd1^–/–^* N2a cells under nonreducing (-β-ME) or reducing (+β-ME) SDS-PAGE. (**F**) Western blot analysis of endogenous POMC processing using ACTH antibody in POMC-expressing mouse pituitary tumor line AtT20 with or without Sel1L. (**G**) Representative confocal images of POMC in POMC-transfected WT and *Sel1L^–/–^* N2a cells. White arrows point to POMC-containing secretory granules, while yellow arrows point to perinuclear POMC. KDEL marks the ER. Representative data from at least 2 independent experiments are shown.

We next explored how Sel1L regulates POMC maturation in the ER using several biochemical approaches. In line with in vivo findings, deletion of either Sel1L or Hrd1 in the neural crest cell line Neuro2A (N2a), a commonly used model system to study POMC trafficking and processing (46), led to a large intracellular accumulation of POMC protein (Figure 5A). Treatment with the proteasomal inhibitor MG132 in WT cells increased POMC protein levels so that they were similar to those in ERAD-deficient cells (Figure 5B). As mRNA levels of transfected POMC were the same among samples in both experiments (Supplemental Figure 6, E and F), these data suggest that Sel1L-Hrd1 ERAD is a major mediator of normal ER POMC protein turnover. Second, HRD1 overexpression in HEK293T cells greatly enhanced POMC polyubiquitination (lanes 1 vs. 2, Figure 5C), which further accumulated following MG132 treatment (lanes 1 vs. 4, Figure 5C). These data suggest that Hrd1 is sufficient to ubiquitinate and target a fraction of POMC for proteasomal degradation. Conversely, loss of HRD1 markedly decreased POMC polyubiquitination below the level of that seen in WT cells (lanes 3 vs. 4, Figure 5D). Of note, polyubiquitinated POMC was only visible in the presence of the proteasomal inhibitor bortezomib (lanes 1 and 2 vs. 3 and 4, Figure 5D), suggesting that ordinarily, ubiquitinated POMC is quickly degraded by proteasomes. Hence, these studies demonstrate that POMC is an endogenous ERAD substrate and that Sel1L-Hrd1 ERAD is responsible for targeting a fraction of POMC molecules for proteasomal degradation.

Notably, ERAD deficiency triggered the formation of high molecular-weight complexes of POMC, which were sensitive to the presence of the reducing agent β-mercaptoethanol (Figure 5E), indicating that in the absence of ERAD, POMC forms high molecular-weight complexes via disulfide bonds. Correspondingly, endogenous POMC processing in a mouse pituitary tumor cell line, AtT20, as demonstrated by the presence of intermediates, was attenuated in the absence of Sel1L (Figure 5F). Finally, to visualize the intracellular trafficking of POMC protein, we performed immunostaining followed by confocal microscopy in N2a cells. In line with previous studies (46, 47), POMC-containing secretory granules were detected throughout the cytoplasm in WT cells. In contrast, ERAD-deficient N2a cells demonstrated predominantly perinuclear staining of POMC (Figure 5G). Together, these data suggest that a fraction of POMC molecules is normally degraded by ERAD and that the absence of ERAD causes POMC to become trapped intracellularly.

### Disease-associated POMC-C28F mutant forms aggregates that escape ERAD.

**Figure 6:**
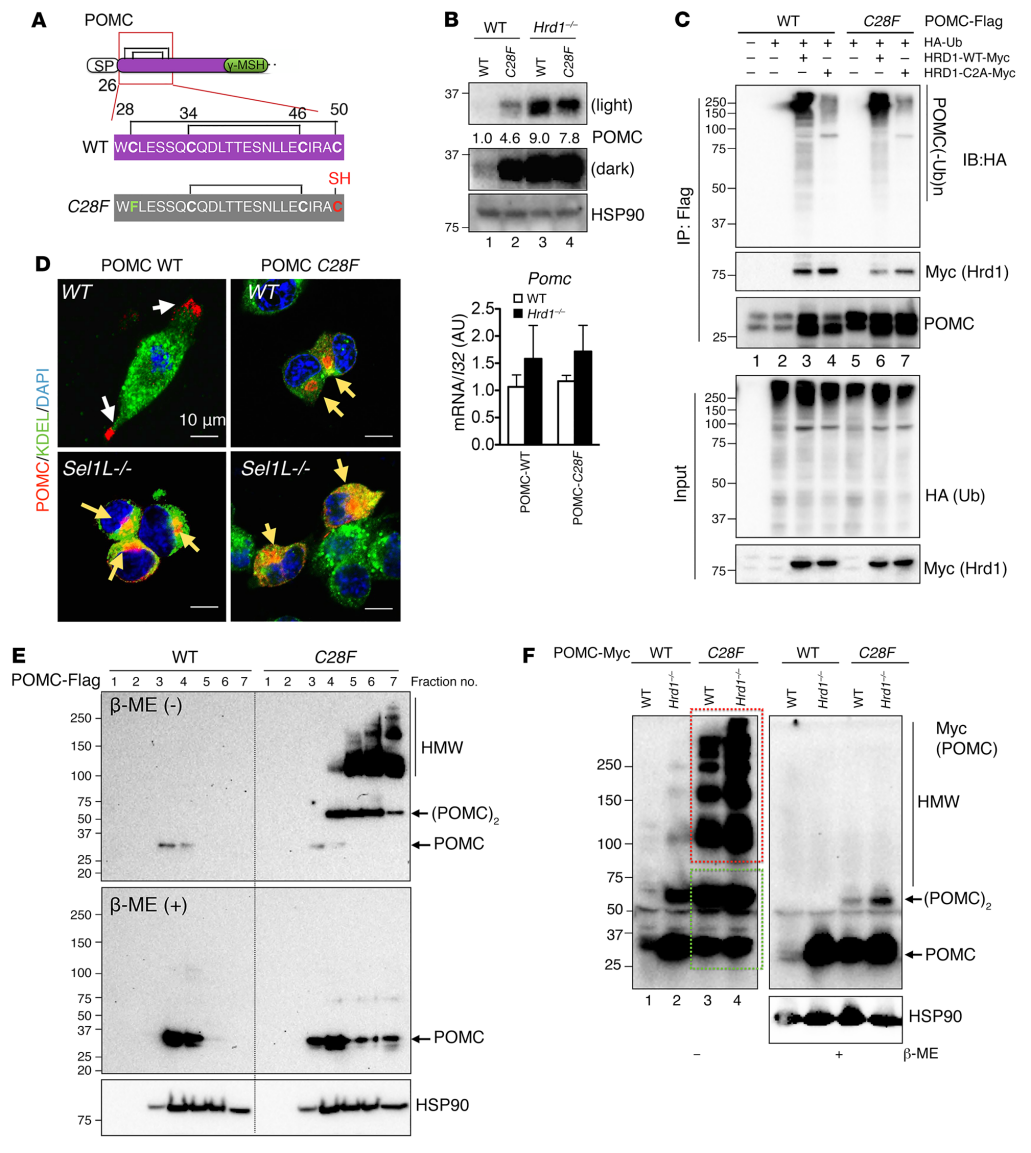
POMC-*C28F* readily forms disulfide bond–mediated aggregates. (**A**) Schematic diagram showing amino acid sequence of POMC 26–50 and the positions of 2 disulfide bonds and a free thiol in POMC-*C28F*. (**B**) Western blot analysis of steady-state levels of POMC proteins in WT and *Hrd1^–/–^* N2a cells transfected with POMC-WT and -*C28F*. mRNA levels of each sample are shown below. (**C**) Western blot analyses of ubiquitination following immunoprecipitation of POMC in HEK293T cells transfected with POMC-WT-Flag or POMC-*C28F*-Flag construct with or without HA-Ub, Myc-tagged HRD1-WT, or HRD1 E3 ligase-dead C2A mutant. (**D**) Representative confocal images of POMC in POMC-transfected WT and *Sel1L^–/–^* N2a cells. White arrows point to secreted POMC in granules, while yellow arrows point to perinuclear POMC, possibly in the form of aggregates. KDEL marks the ER. (**E**) Sucrose gradient fractionation (fractions 1–7 from top to bottom of gradient) of HEK293T cells expressing POMC-WT or -*C28F* under nonreducing (–β-ME) and reducing (+β-ME) SDS-PAGE. (**F**) Western blot analysis of Myc-tagged POMC in WT and *Hrd1^–/–^* N2a cells transfected with POMC-WT or -*C28F* under reducing and nonreducing SDS-PAGE. Red box marks HMW aggregates, while green box marks monomers and dimers. HMW, high molecular weight. (POMC)_2_, POMC dimers. Representative data from at least 2 independent experiments are shown.

The foregoing studies showed that Sel1L-Hrd1–mediated degradation of a fraction of POMC-WT molecules is required for the successful conformational maturation of another fraction of POMC-WT within the ER. To explore the clinical implications of these findings, we investigated the interaction between Sel1L-Hrd1 ERAD and POMC-C28F, a disease-associated mutant (Figure 6A) identified recently in patients with early onset obesity (16). POMC-WT accumulated by 9-fold in ERAD-deficient cells relative to that in WT HEK293T cells (lanes 1 vs. 3, Figure 6B). In contrast, POMC-C28F accumulated by less than 2-fold in ERAD-deficient cells compared with that in WT cells (lanes 2 vs. 4, Figure 6B), suggesting that the effect of ERAD on POMC-C28F protein turnover is less pronounced than that of POMC-WT. Consistently, the basal level of POMC-C28F protein was over 4-fold higher than that of POMC-WT when expressed in WT cells (lanes 1 vs. 2, Figure 6B). mRNA levels of POMC-WT and C28F were similar among the samples (Figure 6B), suggesting that changes in protein levels result from posttranscriptional regulation.

Pointing to the requirement of Sel1L-Hrd1 ERAD in its turnover, POMC-C28F interacted with coexpressed HRD1 and was polyubiquitinated by HRD1 (lanes 5 vs. 6) in a manner that required the E3 ligase activity of HRD1 (lanes 6 vs. 7, Figure 6C). However, upon cycloheximide-mediated (CHX-mediated) translational blockade, POMC-C28F protein decayed more slowly than POMC-WT protein (Supplemental Figure 7A). These data suggest that, while a portion of POMC-C28F may serve as an ERAD substrate, POMC-C28F is not degraded as efficiently as POMC-WT.

Previous studies have suggested that nascent POMC may form 2 disulfide bridges involving C28–C50 and C34–C46 in the ER (47, 48) (Figure 6A). All 4 cysteines are highly conserved from frogs to humans (Supplemental Figure 7B). Like POMC-WT in Sel1L-deficient cells, the POMC-C28F mutant was trapped and accumulated in a perinuclear distribution even in ERAD-competent cells (Figure 6D). Sucrose fractionation assay revealed high molecular-weight aggregates in WT cells expressing POMC-C28F, whereas cells expressing POMC-WT contained predominantly monomers (Figure 6E). A similar pattern of protein aggregation for POMC-C28F was observed using nonreducing SDS-PAGE without prior sucrose fractionation (lanes 3 vs. 1, Figure 6F).

Highlighting the importance of ERAD in POMC-C28F quality control in the ER, ERAD deficiency selectively increased the disulfide-linked aggregation of POMC-C28F into large aggregates without apparent increase of POMC monomers or dimers (lanes 3 vs. 4, Figure 6F). In all of these studies, high molecular-weight complexes of POMC-C28F could be reduced to POMC monomers (and a small population of dimers) upon the addition of β-mercaptoethanol (Figure 6, E and F). Taken together, these data suggest that an important fraction of POMC-C28F mutant evades ERAD and forms high molecular-weight aggregates via aberrant disulfide bond formation.

### Pathogenic POMC-C28F is completely rescued by intragenic suppressor mutation C50S.

**Figure 7:**
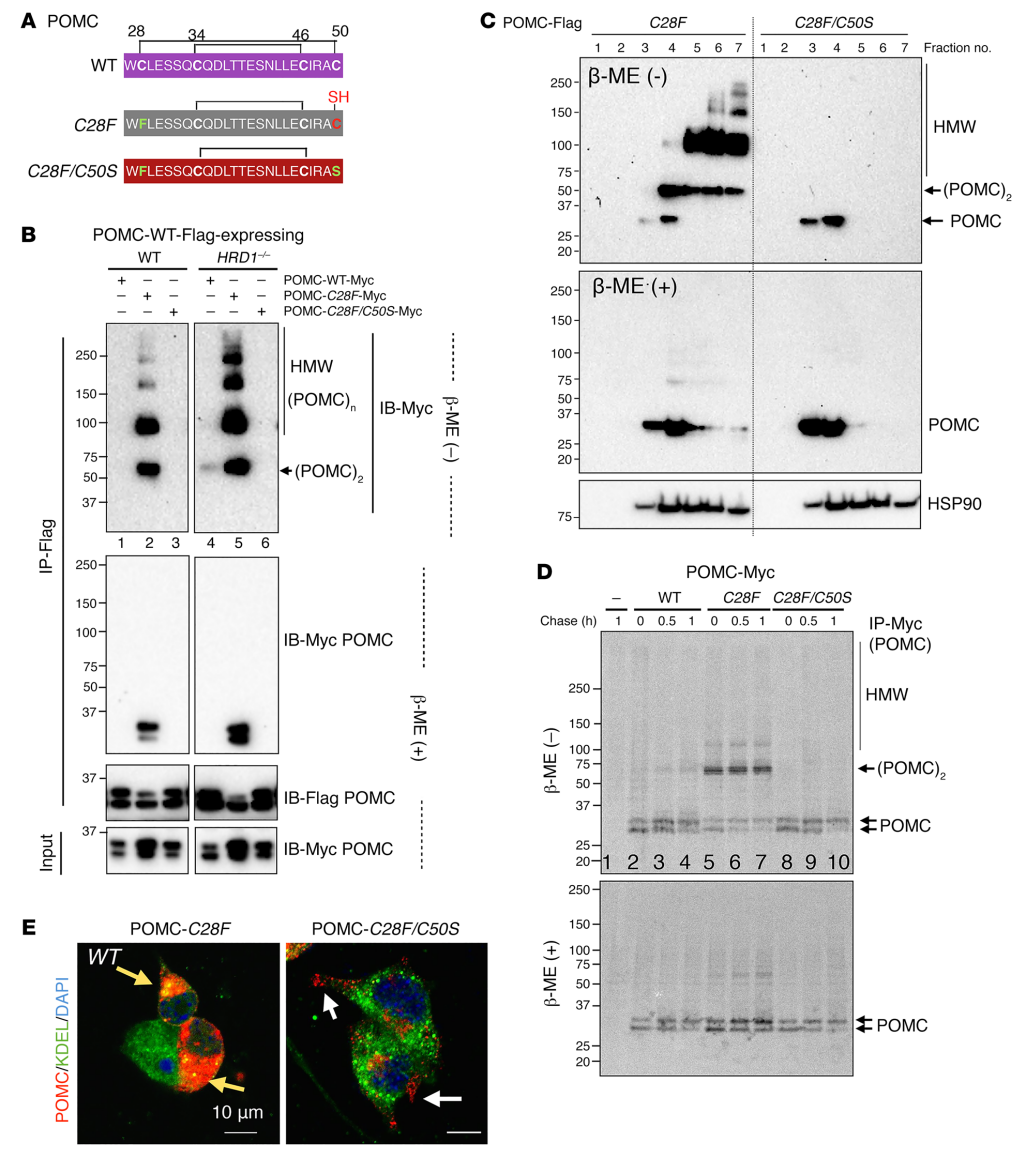
Pathogenic POMC-C28F mutation is completely rescued by an intragenic suppressor mutation C50S. (**A**) Schematic diagram showing the sequence and positions of Cys residues in POMC-WT, C28F, and C28F/C50S. (**B**) Western blot analyses of POMC-Flag immunoprecipitates in HEK293T cells transfected with a combination of Myc- or Flag-tagged POMC under nonreducing (–β-ME) and reducing (+β-ME) SDS-PAGE. Two panels were from the same experiment at the same exposure time, with the irrelevant lanes in the middle cut off. (**C**) Sucrose gradient fractionation and Western blot analysis of HEK293T cells expressing POMC-C28F or -C28F/C50S under nonreducing (–β-ME) and reducing (+ β-ME) SDS-PAGE. (**D**) Metabolic labeling experiments to visualize the maturation of nascent POMC in HEK293T cells transfected with POMC-Myc. Cells were pulsed for 30 minutes and chased for the indicated times, followed by immunoprecipitation with anti-Myc agarose beads and separation on SDS-PAGE gels under nonreducing or reducing conditions and autoradiography. (**E**) Representative confocal images of POMC in POMC-transfected WT N2a cells. White arrows point to secreted POMC in granules, while yellow arrows point to perinuclear POMC, possibly in the form of aggregates. KDEL marks the ER. Representative data from at least 2 independent experiments shown.

We evaluated the possibility that the C to F mutation in C28F (Figure 7A) may destabilize POMC protein due to the bulky side chain of F per se, hence leading to aggregation. We mutated cysteine 28 to serine (C28S) which has side-chain structure similar to that of cysteine, but without the thiol group. However, POMC-C28S formed aberrant disulfide-bonded aggregates similar to those formed by POMC-C28F (lanes 4 vs. 2, Supplemental Figure 7C). We next tested whether the expression of POMC-C28F may disturb the protein maturation of coexpressed POMC-WT in a dominant-negative manner. We coexpressed a combination of Myc- and Flag-tagged POMC and conducted immunoprecipitation to analyze protein aggregation. Indeed, Myc-tagged POMC-C28F formed aberrant high molecular-weight complexes with Flag-tagged POMC-WT in a disulfide bond–dependent manner (lanes 1 vs. 2, Figure 7B). Furthermore, the aberrant interaction and aggregation between POMC-WT-Flag and C28F-Myc worsened in the absence of ERAD (lanes 5 vs. 2, Figure 7B). Strikingly, the aberrant interaction of POMC-C28F-Myc with WT-Flag was abolished when the POMC-C28F was converted to a compound mutant also bearing C50S (lanes 3 vs. 2 and 6 vs. 5, Figure 7B).

To further understand how POMC-C28F/C50S (Figure 7A) abolishes the dominant-negative effect caused by C28F alone, we analyzed the self-aggregation ability of POMC-C28F/C50S. Addition of the C50S mutation completely abolished POMC-C28F aggregation, as demonstrated using either sucrose gradient analysis (Figure 7C) or nonreducing SDS-PAGE without fractionation (lanes 3 vs. 2, Supplemental Figure 7C and lanes 1 and 2 vs. 3 and 4, Supplemental Figure 8A), resulting in monomers similar to those seen for POMC-WT in WT cells (Figure 6E). The C50S mutation also abolished disulfide bond–mediated aggregation of POMC-C28S (lanes 5 vs. 4, Supplemental Figure 7C).

We next performed a pulse-chase experiment to visualize folding and maturation of newly synthesized POMC. Newly synthesized POMC-WT protein existed predominantly as monomers (lanes 2–4, Figure 7D). In contrast, nascent POMC-C28F formed mainly dimers and higher molecular-weight aggregates (lanes 5–6, Figure 7D), which was reversed by the additional intragenic C50S mutation.

Unlike POMC-C28F which aggregated in both WT and ERAD-deficient cells (Figure 6F), POMC-C28F/C50S did not aggregate even in ERAD-deficient cells (lanes 3 vs. 1, Supplemental Figure 8A). Confocal microscopy further revealed the anterograde transport of POMC-C28F/C50S to secretory granules in WT N2a cells in contrast with the perinuclear aggregation of POMC-C28F (Figure 7E) — indicating successful escape from ER quality control, leading to secretion of POMC-C28F/C50S. This observation is further supported by a previous study showing that the C28–C50 disulfide bridge is not required for trafficking of POMC to secretory granules (47). Finally, following translation blockade by CHX treatment, the intracellular levels of POMC-C28F/C50S protein declined faster than those of POMC-C28F (Supplemental Figure 8B), which is likely correlated with successful egress of POMC-C28F/C50S from the ER. Taken together, these studies demonstrate that an unpaired C50 residue renders POMC-C28F highly reactive to form aggregates via improper intermolecular disulfide bonds.

## Discussion

Peptide hormones are key regulators of mammalian physiology, and disruptions in hormone production and secretion are key pathogenic events in many human diseases. Prohormone synthesis begins in the ER, where essential steps of protein folding take place. If the polypeptides meet ER quality control, they progress through anterograde transport to distal compartments of the secretory pathway. Here, we report a surprising finding that ERAD involving the Sel1L-Hrd1 protein complex is required for POMC maturation in the ER, which, when defective, upregulates food intake, leading to age-associated obesity as a result of intracellular retention of POMC. This study points to a previously unappreciated role of ERAD in POMC neurons in prohormone maturation within the ER, by which it regulates feeding behavior, energy homeostasis, and obesity. Moreover, our data demonstrate that the pathogenic C28F mutation triggers POMC protein aggregation due to the presence of an unpaired cysteine at position 50.

**Figure 8:**
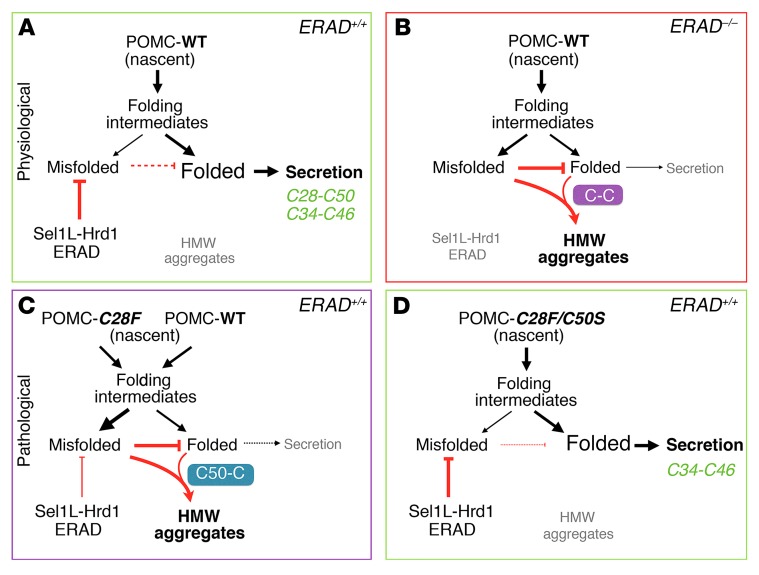
The role of Sel1L-Hrd1 ERAD in POMC maturation within the ER under (patho-)physiological conditions. (**A**) Under physiological conditions, Sel1L-Hrd1 ERAD plays an important role in promoting a conducive environment for the maturation of nascent POMC in the ER by degrading, likely misfolded, POMC. (**B**) In the absence of ERAD, misfolded POMC accumulates and interferes with the maturation of nascent POMC in the ER in a disulfide bond–dependent manner. (**C**) Under pathological conditions, POMC-*C28F* is consistently misfolded and forms aggregates via intermolecular C50-mediated disulfide bonds. This model explains the dominant-negative effect of POMC-*C28F*. (**D**) The maturation defects of POMC-*C28F* can be completely rescued by an intragenic suppressor mutation, *C50S*.

We found that a significant number of POMC molecules — presumably the misfolded cohort — are an obligatory ERAD substrate, as this degradative activity provides an ER environment conducive to the remaining population of POMC molecules attaining a proper conformation (Figure 8A). While it is not surprising that ERAD degrades a fraction of newly synthesized unstable POMC, it was unexpected that ERAD deficiency caused ER retention and aggregation of a large proportion of all POMC proteins in vivo (Figure 8B). This, rather than ER stress–mediated cell death, appears to trigger the downstream deficiency of POMC-derived peptides under conditions of ERAD deficiency.

Furthermore, our data demonstrate that the dominant-negative effect of the disease-associated POMC-C28F mutant is mediated through a highly reactive free thiol group at residue C50, which promotes POMC aggregation (Figure 8C). Strikingly, the maturation defects of POMC-C28F can be completely rescued by intragenic suppressor mutation C50S (Figure 8D).

*Sel1L^POMC^* mice develop obesity accompanied by impaired leptin response in the POMC neurons, but the primary defect in the absence of ERAD appears to involve defective POMC processing in the ER. Many POMC-derived peptide hormones are diminished in the axons of POMC neurons, while the POMC prohormone accumulates intracellularly. Our data suggest that ERAD of nascent POMC proteins, presumably in the misfolded conformation, is a prerequisite for maturation and exit of other POMC molecules from the ER, acting by preventing protein aggregation through aberrant disulfide bonds (Figure 8, A and B). Importantly, our *Sel1L^POMC^* mice developed obesity around 15 weeks rather than earlier, as is seen in POMC-deficient mice (9, 13–15). The delay in onset of obesity suggests that impaired Sel1L-Hrd1 ERAD function attenuates, rather than completely blocks, POMC maturation within the ER, and thus, more time is required to develop the obesity phenotype. Studies are underway to explore other possible defects in the steps of protein trafficking and proteolytic processing in Sel1L-deficient POMC neurons.

The effect of POMC-specific ERAD clearly does not involve ER stress-mediated inflammation or cell death. Indeed, *Xbp1* mRNA splicing and levels of other UPR markers were very low in arcuate neurons of both young and old *Sel1L^POMC^* mice, as were several other markers of UPR. We specifically looked for both inflammation and increased cell death in *Sel1L^POMC^* neurons and could find no evidence of either. It should be noted that the cohorts used in this study were fed a LFD rather than a HFD, which may help explain the lack of ER stress and inflammation in our model. Indeed, ER stress has emerged as a critical link in the development of leptin and insulin resistance in neurons of animals with HFD-induced obesity. HFD may induce ER stress in arcuate POMC neurons, which may alter POMC processing and trafficking (49, 50). Two recent studies of the role of the UPR sensor IRE1α in POMC neurons showed mixed results in terms of susceptibility to diet-induced obesity (25, 26). It is worth pointing out that, unlike *Sel1L^POMC^*-KO mice, *Ire1a^POMC^*-KO mice do not develop age-associated obesity on a LFD (25, 26). Hence, this study sheds light on early events associated with POMC maturation under normal physiology. Further studies are required to investigate the relative contributions of ERAD and UPR in POMC neurons in the context of diet-induced obesity.

This study implies a therapeutic potential for targeting ERAD in diseases associated with prohormone conformational maturation defects. Our data show that Sel1L-Hrd1 ERAD can degrade POMC-WT and, to a lesser extent, disease-associated POMC-C28F. We speculate that the POMC-C28F mutant is less efficiently targeted for proteasomal degradation because it more readily forms high molecular-weight aggregates that sequester it from the Sel1L-Hrd1 degradative machinery. Unexpectedly, our data further reveal that the formation of protein aggregates of POMC-C28F is mediated via aberrant intermolecular disulfide bonds between unpaired C50 and another free cysteine (Figure 8C). When C50 is mutated to serine as in POMC-C28F/C50S, protein aggregation is abolished and normal protein turnover and secretion seem to be recovered (Figure 8D). The requirement of C50 in the formation of protein aggregates of C28F mutant is very interesting and reminiscent of observations of the *Akita* mutant proinsulin causing diabetes mellitus (51, 52).

In a recent study, we reported that proAVP, the ER precursor for the antidiuretic hormone AVP, also requires ERAD activity of the Sel1L-Hrd1 protein complex for its conformational maturation, and mice with inducible or AVP neuron-specific *Sel1L* deletion develop central diabetes insipidus, a condition caused by AVP deficiency (39). Similarly to what occurred in the present study, ERAD deficiency was found to cause ER retention and aggregation of a large proportion of all proAVP protein via inappropriate intermolecular disulfide bonds. Taking these data together with the current report, we posit that ERAD serves a critical role in maintaining a suitable environment for efficient prohormone folding in the ER. It will be interesting to learn whether other physiologically important prohormones such as proinsulin, pro-oxytocin, and pro–agouti-related protein (pro-AGRP) also require Sel1L-Hrd1 ERAD activity for their ER folding and maturation.

The most striking feature of this study, in addition to our most recent study on proAVP (39), is the profound effect of ERAD efficiency on prohormone conformational maturation that leads to disease. While it is largely predictable that ERAD degrades a fraction of newly synthesized unstable prohormones, our data reveal a role for ERAD in creating a “safe” environment for nascent prohormones to reach proper conformation without the distraction of aggregation. In the case of disease mutants, they may readily form disease-associated aggregates that are resistant to ERAD, causing a loss-of-function phenotype. The delineation of the role of ERAD in effectively regulating feeding behavior and obesity development via monitoring the correct conformational maturation of both WT and mutant POMC within the ER may serve as a prototype for understanding many diseases associated with prohormone maturation defects and highlight the importance of targeting ERAD in disease therapy.

## Methods

### Mice.

*Sel1L^fl/fl^* mice on the C57BL/6J background (29) were crossed with mice on the POMC-promoter–driven Cre line on the C57BL/6J background (B6.FVB-Tg(Pomc-cre)1Lowl/J, JAX 010714) (42) to generate POMC-specific Sel1L-deficient mice (*Sel1L^POMC^*) and control littermates (*Sel1L^fl/fl^*). In addition, we crossed *Sel1L^POMC^* mice with Pomc-eGFP reporter mice on the C57BL/6J background (C57BL/6J-Tg(POMC-EGFP)1Low/J, JAX 009593) (13) to generate *Sel1L^POMC^;POMC-eGFP* (*Sel1L^fl/fl^;Pomc-Cre;POMC-eGFP*) and control littermates *Sel1L^POMC/+^;POMC-eGFP* (*Sel1L^fl/+^;Pomc-Cre;POMC-eGFP*). WT B6 mice were purchased from the Jackson Laboratory and bred in our mouse facility. Mice were fed a LFD (13% fat, 67% carbohydrate, and 20% protein, Harlan Teklad 2914).

### General feeding behavior and restricted feeding.

For daily food intake measurement, mice were single housed. Food intake was measured by weighing leftover food pellets. The bottom of the cage was searched to include all leftover food. Thirty-week-old *Sel1L^POMC^* mice were split into 2 groups. One group had continuous free access to food, whereas the other group was fed 1 g at the start of light hours and 2.2~2.5 g at the start of dark hours, totaling approximately 3.2 to 3.5 g/d. At 28 days, free access to food was reintroduced to the latter group.

### Leptin and α-MSH treatment.

Eight-week-old mice were injected i.p. with 0.9% saline followed by leptin (2 mg/kg body weight, R&D Systems, 498-OB-05M) or directly with α-MSH Ac-SYSMEHFRWGKPV-NH2 at 1 mg/kg body weight (Gene Script Inc., RP10644) 1 hour before the onset of the dark cycle for 3 consecutive days. Body weight and food intake were monitored daily during the treatment period. Brains were collected using perfusion fixation 30 minutes after the last leptin injection on day 3.

### Tissue and blood collection.

Blood was collected from anesthetized mice through cardiac puncture and then transferred to microcentrifuge tubes. Collected blood was kept at room temperature for 30 minutes prior to centrifugation at 3,000 *g* for 15 minutes at 4°C. Serum was aliquoted and stored at −80°C until analysis. To collect brain tissue enriched for the ARC region, the brain was dissected using an Adult Mouse Brain Slicer Matrix (BSMAA001-1, Zivic Instruments) and further microdissected under light microscope. Immediately after collection, brain tissue and peripheral tissues for Western blot or quantitative PCR (qPCR) analysis were snap-frozen in liquid nitrogen. Frozen tissues were stored at −80°C.

### α-MSH ELISA.

Hypothalamus was harvested from mice fed and ad libitum LFD and lysed in lysis buffer (150 mM NaCl, 100 mM Tris-Cl pH 7.5, 1% Triton-X 100, 1 mM EDTA, 1 mM EGTA with protease inhibitor). α-MSH levels in the hypothalamus were measured using the α-MSH EIA Kit (EK-043-01, Phoenix Pharmaceuticals). This kit has no crossreactivity toward ACTH, cocaine- and amphetamine-regulated transcript protein (CART), AGRP, and leptin per the manufacturer.

### Preparation of brain sections.

Mice were fixed via transcardial perfusion with 4% paraformaldehyde (PFA) (19210, Electron Microscopy Sciences) as previously described (53). Briefly, mice were anesthetized with isoflurane and euthanized via decapitation. Brain was then postfixed in 4% PFA for 2 hours at 4°C, dehydrated in 15% sucrose overnight at 4°C, and sectioned on a cryostat (Microm HM550 Cryostat, Thermo Fisher Scientific). Brain sections (30 μm) were stored in DEPC-containing anti-freezing media (50% 0.05 M sodium phosphate pH 7.3, 30% ethylene glycol, 20% glycerol) at −20°C. Different brain regions were identified using the Paxinos and Franklin atlas (54). Counted as distance from bregma, the following coordinates were used: PVN (–0.82 mm to –0.94 mm) and ARC (–1.58 mm to –1.7 mm).

### Antibodies used for Western blot, immunostaining, and immunohistochemistry.

Antibodies for Western blot were as follows: Sel1L (rabbit, 1:2,000; catalog ab78298, Abcam), BiP/GRP78 (goat, 1:1,000, catalog sc-1051, Santa Cruz Biotechnology Inc.), HSP90 (rabbit, 1:5,000; catalog sc-7947, Santa Cruz Biotechnology Inc.), FLAG (mouse, 1:2000; catalog F-1804, Sigma-Aldrich), MYC (rabbit, 1:2000; catalog C3956, Sigma-Aldrich), IRE1α (rabbit, 1:2,000; catalog 3294, Cell Signaling Technology), p-eIF2α (rabbit, 1:2000; catalog 3597, Cell Signaling Technology), eIF2α (rabbit, 1:2000; catalog 9722, Cell Signaling Technology), p-JNK (mouse, 1:2000; catalog 9255, Cell Signaling Technology), JNK (rabbit, 1:1000, catalog sc-571, Santa Cruz Biotechnology Inc.), and ACTH (rabbit, 1:2000, catalog G-001-06, Phoenix Pharmaceuticals). Hrd1 antibody (rabbit, 1:200) was provided by Richard Wojcikiewicz (State University of New York Upstate Medical University, Syracuse, New York, USA). ACTH antibody (rabbit, 1:2000) was previously described (12, 55). Secondary antibodies for Western blot were goat anti-rabbit IgG HRP and goat anti-mouse IgG HRP at 1:5,000, both from Bio-Rad. Donkey anti-goat IgG HRP was from Jackson ImmunoResearch Laboratories. See complete unedited blots in the supplemental material.

Antibodies for immunostaining were as follows: HA (mouse, 1:500; catalog H9658, Sigma-Aldrich), XTP3-B (goat, 1:500; catalog sc-161409, Santa Cruz Biotechnology Inc.) and Hrd1 (rabbit, 1:500), POMC (goat, 1:250; catalog NB100-1533, Novus Biologicals), ACTH (rabbit, 1:100; catalog G-001-06, Phoenix Pharmaceuticals), α-MSH (sheep, 1:2,000; catalog AB5087, Millipore), β-endorphin (rabbit, 1:2,000; catalog H-022-33, Phoenix Pharmaceuticals, provided by Carol Elisa), and GFP (chicken, 1:500; catalog GFP-1020, Aves Labs). Antibodies for immunohistochemistry were as follows: Hrd1 (rabbit, 1:1000) and p-Y705 STAT3 (rabbit, 1:1,000; catalog 9145, Cell Signaling Technology).

Secondary antibodies for fluorescent immunostaining and immunohistochemistry (all 1:500) were anti-mouse IgG Cy3; anti-rabbit IgG Alexa Fluor 594 and Alexa Fluor 647; anti-goat IgG Alexa Fluor 594; anti-sheep IgG Cy5; and anti-rabbit IgG Biotin (Jackson ImmunoResearch). Goat anti-chicken IgY FITC (1:500) was from Aves Labs.

### Immunofluorescent staining and immunohistochemistry.

For immunohistochemistry, free-floating brain sections were permeabilized by 0.3% Triton X-100 for 10 minutes at room temperature and then incubated in blocking solution (1% donkey serum, 0.03% Triton X-100 in 0.05 M potassium PBS [K-PBS]) for 30 minutes at room temperature. After blocking, free-floating brain sections were incubated with primary antibodies overnight at 4°C and, following 3 washes with K-PBST (0.03% Triton X-100 in 0.05M K-PBS), were incubated with secondary antibodies for 1 hour at room temperature followed by incubation with avidin-biotin complex (PK-4000, Vector Laboratories). DAB (SK-4100, Vector Laboratories) was used for colorimetric detection, and brain sections were then mounted on gelatin-coated slides (SLD01-CS, Southern Biotech). After dehydration with xylene and ethanol, coverslip (Fisherfinest Premium Cover Glasses, 12-548-5P, Fisher Scientific) was placed with Permount (SP15-100, Fisher Scientific). For fluorescent immunostaining, free-floating brain sections were simultaneously incubated with primary antibodies in blocking buffer (0.3% donkey serum and 0.25% Triton X-100 in 0.1 M PBS) overnight at room temperature. Following 3 washes with PBS, sections were sequentially incubated with secondary antibodies for 2 hours at room temperature. Brain sections were then mounted on gelatin-coated slides (SLD01-CS, Southern Biotech). Counterstaining and mounting were performed with mounting medium containing DAPI (H-1200, Vector Laboratories) and Fisherfinest Premium Cover Glasses (12-548-5P, Fisher Scientific). For immunostaining in N2a cells, 12 mm diameter cover glass (72230-01, Electron Microscopy Sciences) was coated by 0.1% poly-l-lysine solution (MilliporeSigma, P8920). Cells (2 × 10^4^) were placed on coated cover glass in a 24-well plate and then transfected with POMC constructs using Lipofectamine 2000. Twenty-four hours after transfection, cells were fixed by 4% formaldehyde (89370-094, VWR) for 10 minutes at room temperature, washed by K-PBS, and permeabilized using 0.3% Triton X-100 for 10 minutes at room temperature followed by incubation in blocking solution. Cells were stained as described above.

### Signal quantitation.

To quantify immunoreactivity, identical acquisition settings were used for imaging each brain section from all groups within an experiment. The numbers of immunoreactivity-positive soma analysis and intensity of immunoreaction were quantified in 3D stack volumes after uniform background subtraction using NIS Elements AR software (Nikon) and FIJI (NIH).

### CRISPR/Cas9-based gene editing.

Generation of HRD1-deficient HEK293T cells was previously described (29). Single guide RNA (sgRNA) oligos for human HRD1, 5′-GGACAAAGGCCTGGATGTAC, were used. To generate Sel1L- and Hrd1-deficient mouse N2a cells and Se1L-deficient mouse AtT20 cells, sgRNA oligo for mouse Sel1L (GCCAGCAACTACTTTGCCCG) or mouse Hrd1 (ATCCATGCGGCATGTCGGGC) was inserted into lentiCRISPR v2 (Addgene plasmid 52961). We used third generation lentiviral packaging plasmids (RRE [gag/pol], VSV-G, and REV) to deliver CRISPR constructs into the cells. Twenty-four hours after infection, cells were cultured in medium containing 2 μg/ml puromycin for 24 hours and then in normal growth medium.

### Plasmids.

To construct mouse POMC plasmids, cDNA was synthesized from mouse hypothalamus total RNA using reverse transcriptase (18080-085, Invitrogen) per the manufacturer’s protocol. The *Pomc* coding region was amplified by PCR using a primer set (5′-CCCAAGCTTACCATGCCGAGATTCTGCTACAGT-3′, 5′-CCGCTCGAGTCACTGGCCCTTCTTGTGCGC-3′) and inserted into pcDNA3.1(+). For construction of POMC point mutants and C-terminal tagged constructs, quick change mutagenesis was performed using PFU DNA polymerase (600140, Agilent). The following primers were used for mutagenesis: POMC-C28F (5′-GATGTGTGGAGCTGGTTCCTGGAGAGCAGCCAG-3′, 5′-CTGGCTGCTCTCCAGGAACCAGCTCCACACATC-3′), POMC-C50S (5′-CTGCTGGCTTGCATCCGGGCTTCCAAACTCGACCTCTCGCTGGAG-3′, 5′-CTCCAGCGAGAGGTCGAGTTTGGAAGCCCGGATGCAAGCCAGCAG-3′), and C-terminal Flag-tagged constructs (5′-GAACGCGCACAAGAAGGGCCAGGACTACAAAGACGATGACGACAAGTGACTCGAGTCTAGAGGGCCC-3′, 5′-GGGCCCTCTAGACTCGAGTCACTTGTCGTCATCGTCTTTGTAGTCCTGGCCCTTCTTGTGCGCGTTC-3′). Human HRD1 WT and ring-finger mutant C2A (C291A/C294A) constructs were provided by Yihong Ye (National Institute of Diabetes and Digestive and Kidney Diseases, Bethesda, Maryland, USA). pcDNA3-HA-Ub was provided by Hideki Nishitoh (University of Miyazaki, Miyazaki, Japan). POMC-Myc was provided by Martin Spiess (University of Basel, Basel, Switzerland).

For further information, see Supplemental Methods.

### Statistics.

Results are expressed as mean ± SEM unless otherwise stated. Statistical analyses were performed with GraphPad Prism (GraphPad Software Inc.). Comparisons between 2 groups were made by unpaired 2-tailed Student’s *t* test. Two-way ANOVA followed by Bonferroni’s post-test was used to determine statistical significance for more than 2 groups with 2 factors. *P* < 0.05 was considered statistically significant. All experiments were repeated at least twice or performed with several independent biological samples, and representative data are shown.

### Study approval.

All animals experiments were approved by Cornell University Institutional Animal Care and Use Committee, Ithaca, New York, USA (#2007-0051) and University of Michigan Institutional Animal Care and Use Committee (#PRO00006888) guidelines, Ann Arbor, Michigan, USA.

## Author contributions

GHK designed and performed most of the in vivo and in vitro experiments. GS designed and performed several biochemical studies. LH, DRMS, and SS provided technical assistance. QL provided reagents. EAN, MJL, and MGM provided reagents and discussions. PA provided discussions and edited the manuscript. LQ designed experiments and directed the study. LQ and GHK wrote the manuscript. All other authors approved the manuscript.

## Supplementary Material

Supplemental data

## Version 1 02/19/2018

Electronic publication

## Version 2 03/01/2018

Print issue publication
