# Supplementary material for: Hypothalamic ER–associated degradation regulates POMC maturation, feeding, and age-associated obesity
Source: J Clin Invest. 2018 Feb 19;128(3):1125–40. doi: 10.1172/JCI96420 (PMC5824855; doi:10.1172/JCI96420)
Supplement: Supplemental data [file jci-128-96420-s001.pdf]

Supplementary materials

Hypothalamic ER-associated degradation regulates POMC maturation, feeding and age-associated obesity

Geun Hyang Kim[1], Guojun Shi[1], Diane R. M. Somlo[2,6], Leena Haataja[5], Soobin Song[2], Qiaoming Long[3], Eduardo A. Nillni[4], Malcolm J. Low[1,5], Peter Arvan[1,5], Martin G. Myers, Jr[1,5], and Ling Qi[1,5]

Supplementary figures 1-8

Supplementary methods and references

Supplementary Figures and Legends

Figure S1. Expression of Sel1L-Hrd1 ERAD in the ARC is responsive to physiological signals.
(A) Representative images of negative control IgG in the brains of 7-week-old C57BL/6J mice on LFD (n=2 each group, two repeats). (B-C) Gene expression in the ARC of 7-week-old C57BL/6J mice after fasting-refeeding condition (n=2-3 each group, two repeats) (B) or injected with leptin for 6 hours while fasting (n=2-4 each group) (C). Values, mean ± SEM, *, p<0.05; **, p<0.01 by one-way ANOVA (B) or Student's *t* test (C).

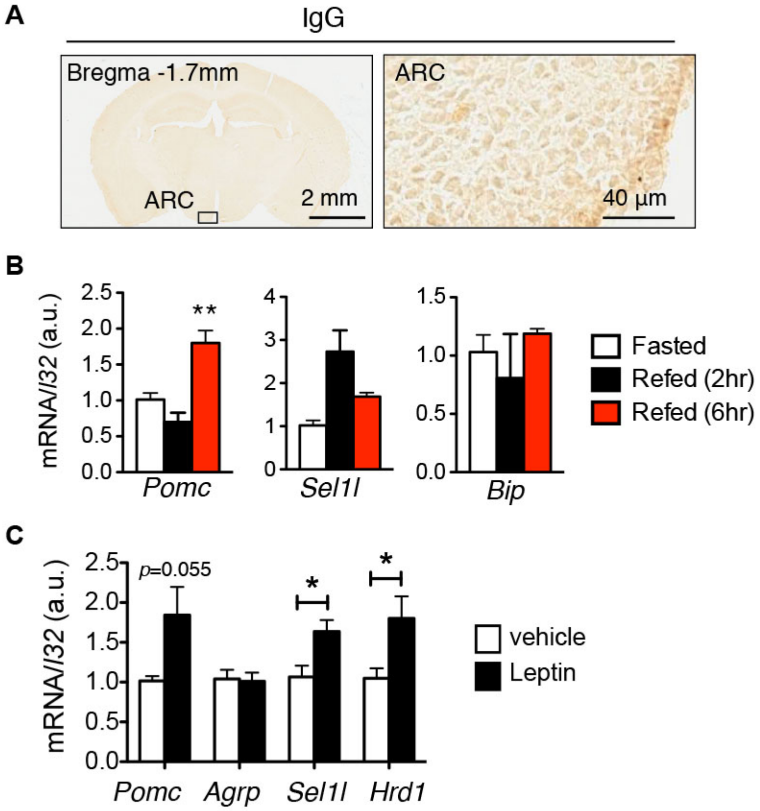

Figure S2. *Sel1L^{POMC}* mice develop age-associated obesity.
(A) Images of H&E stained peripheral tissues from 10-week-old mice on LFD (n=3 each group). (B) Body compositions of 40-week-old cohorts (*Sel1L$^{f/f}$, Sel1L$^{POMC/+}$, Sel1L^{POMC}*) on LFD. (C) Representative image of inguinal (iWAT) and pancreatic islet of 40-week-old cohorts on LFD. Values, mean ± SEM. *n.s.*, not significant; *, p<0.05; **, p<0.01; ***, p<0.001 by two-way ANOVA (A).

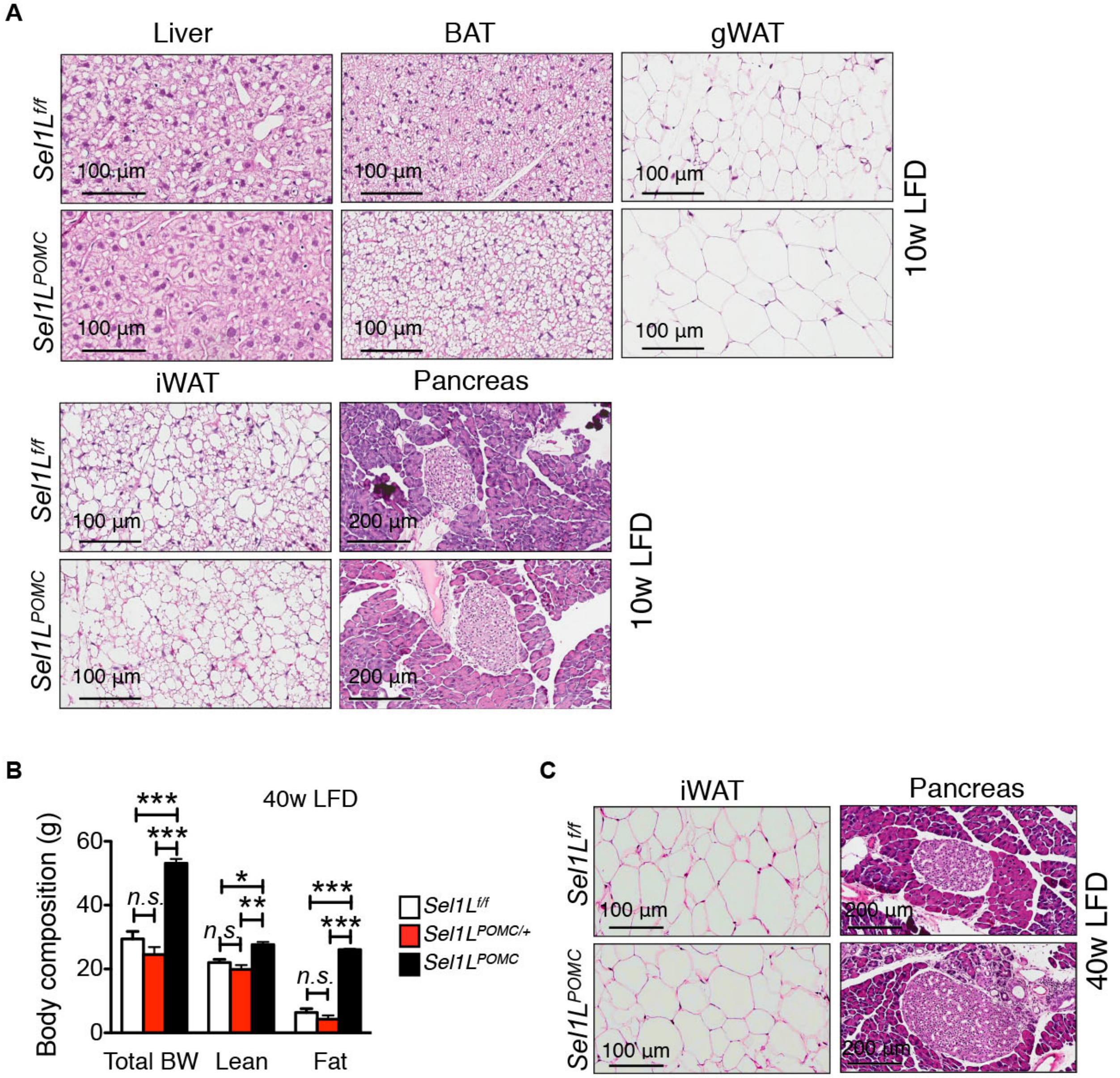

Figure S3. Glucose and insulin-tolerance tests (GTT/ITT).
(A) Body weight of mice used for GTT and ITT. (B-E) GTT (B,D) and ITT (C,E) in 8~12-week-old mice (B-C) and 20~23-week-old mice (D-E) where mice were fasted for 16 or 6 hours prior to glucose (2 mg/kg body weight) or insulin (1 unit/kg body weight) injection, respectively. n=4-6 each group, two repeats. Values, mean ± SEM. *n.s.*, not significant; *, p<0.05; ***, p<0.001 by two-way ANOVA.

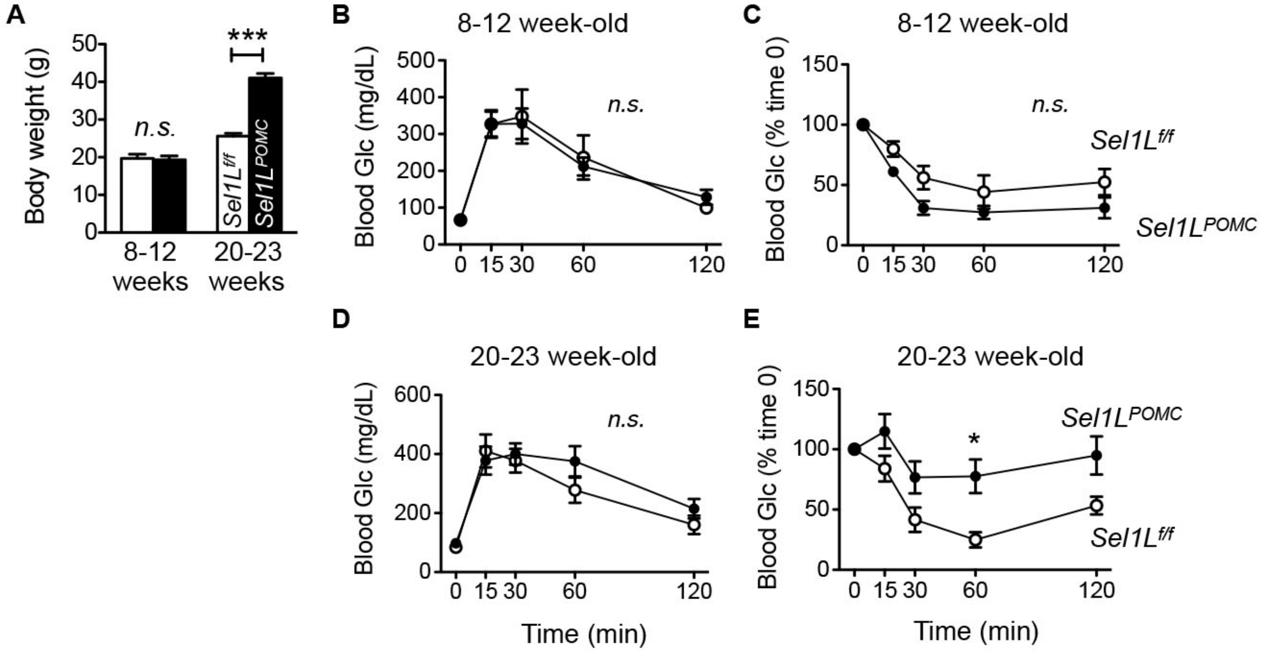

Figure S4. Metabolic rate and physical activity analyses of young and old cohorts on LFD.
O_2 consumption, CO_2 production (A,C), respiratory exchange ratio (RER) and physical activity (B,D) of 8-week-old (A,B) and 40-week-old (C,D) mice fed ad libitum LFD, respectively. n=3-4 mice each group. Values, mean ± SEM. *n.s.*, not significant by two-way ANOVA.

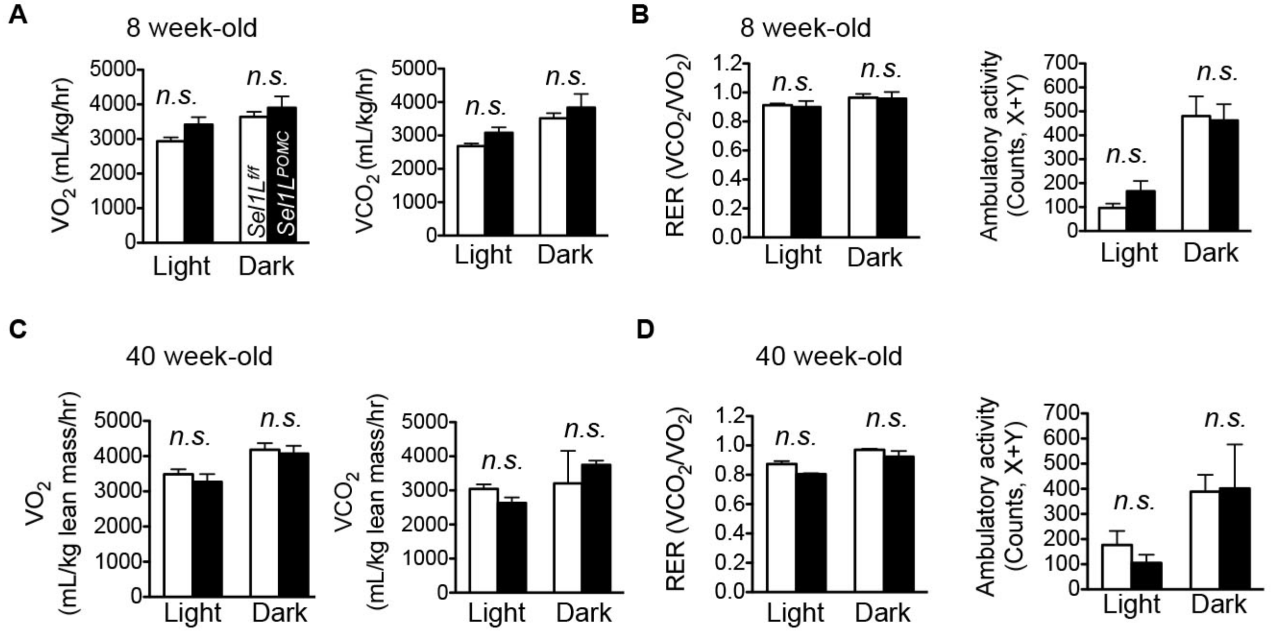

Figure S5. Sel1L deficiency in POMC neurons is not associated with elevated inflammation and cell death in the ARC.

(A) UPR analysis of the ARC: phosphorylated IRE1α using phos-tag (P-T) and total protein levels of BiP and p-eIF2α, with quantitation shown in B (n=2 each group). (C-D) Q-PCR analysis of inflammatory genes in the ARC of 8- (C) and 40-week-old (D) mice (n=2 each group). No gene expression analyzed was significantly different. (E) TUNEL analysis in the ARC of 40-week-old mice (n=5 each group), with a positive control shown in F, where brain sections were treated with DNase I prior to TUNEL staining. (G) Quantitation of TUNEL positive cells and total cells in the ARC. (H) Western blot analysis of Caspase 3 in ARC of 8- and 40-week-old mice (n=2 each). Colon isolated from mice treated with 3% dextran sodium sulfate (DSS) was used as a positive control. Values, mean ± SEM. *n.s.*, not significant; *, p<0.05; **, p<0.01 by two-way ANOVA (B,G) and Student's *t* test (C,D).

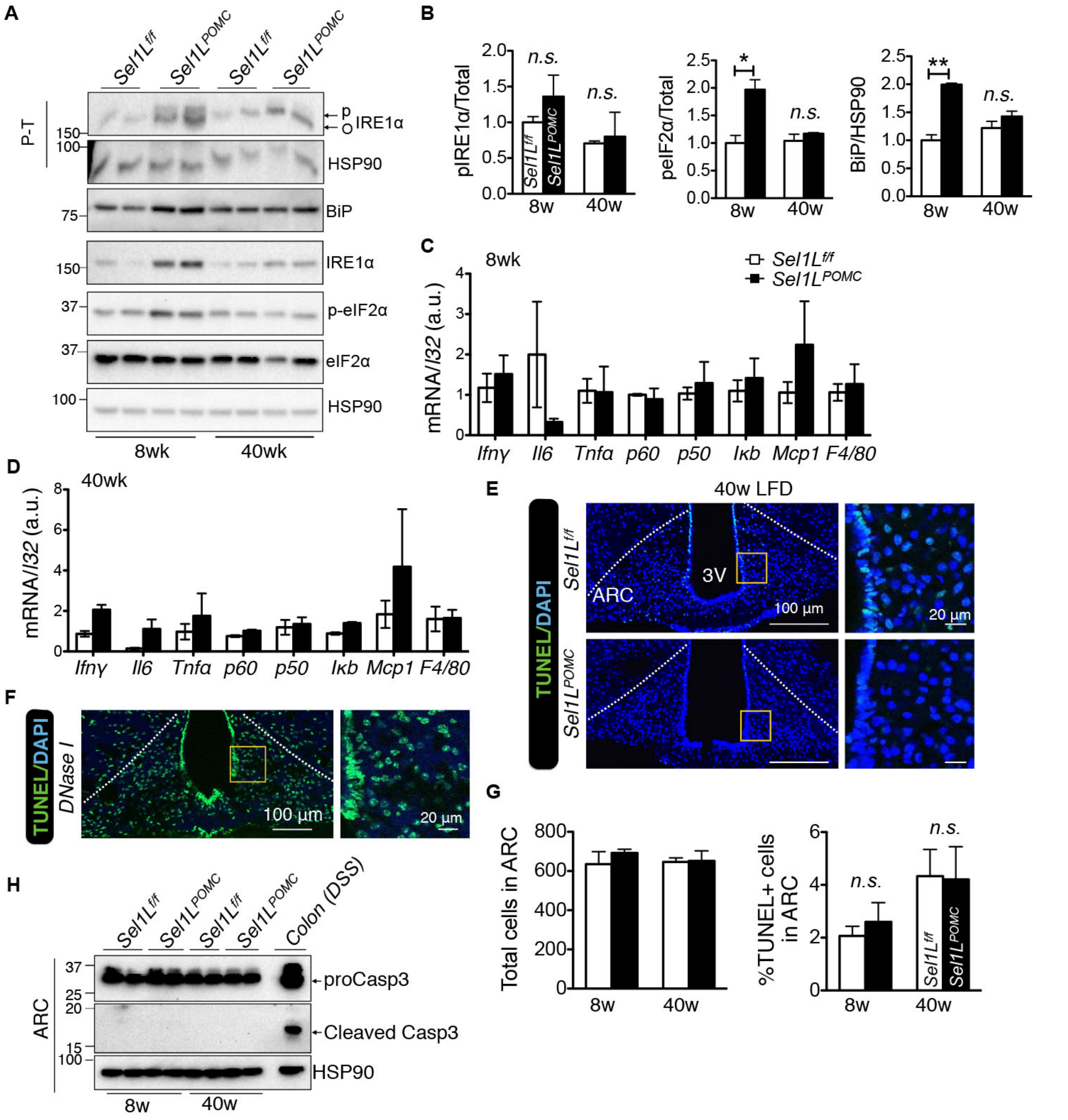

Figure S6. ER retention of POMC in the absence of Sel1L.
(A) Quantitation of POMC prohormone signal intensity in cell body and neuronal axon in the ARC of 5~10-week-old wildtype (N=79 cells of 5 mice) and *Sel1L*^{POMC}mice (N=289 cells of 6 mice). (B) Average signal intensities of α-MSH in neuronal axon terminals in PVN of 5~10-week-old mice (n=4 mice each), β-endorphin (n=4-5 mice each) and ACTH (n=2-3 mice each) in cell body and neuronal axons in ARC and DMH of 5~8-week-old mice fed ad libitum LFD. A total of 15-17 image layers were compressed, from which signal intensity from ~2000 signal particles were counted. Representative images are shown in Figure 4C-E. (C-D) Representative immunofluorescent images of β-endorphin (red) and ER chaperone XTP3-B (green) in the ARC of control *Sel1L*$^{f/f}$ (C) and *Sel1L*^{POMC} (D) mice (n=2-3 each group, two repeats). The lower and side panels show zoomed-in images in the x-y, x-z and y-z axes of β-endorphin in the cell co-stained with XTP3-B. (E-F) mRNA levels of various N2a cells transfected with POMC, with or without MG132. These data correspond to Western blot data shown in Figure 5A (E) and 5B (F). Values, mean ± SEM. *, p<0.05; **, p<0.01; ***, p<0.001 by Student's *t* test.

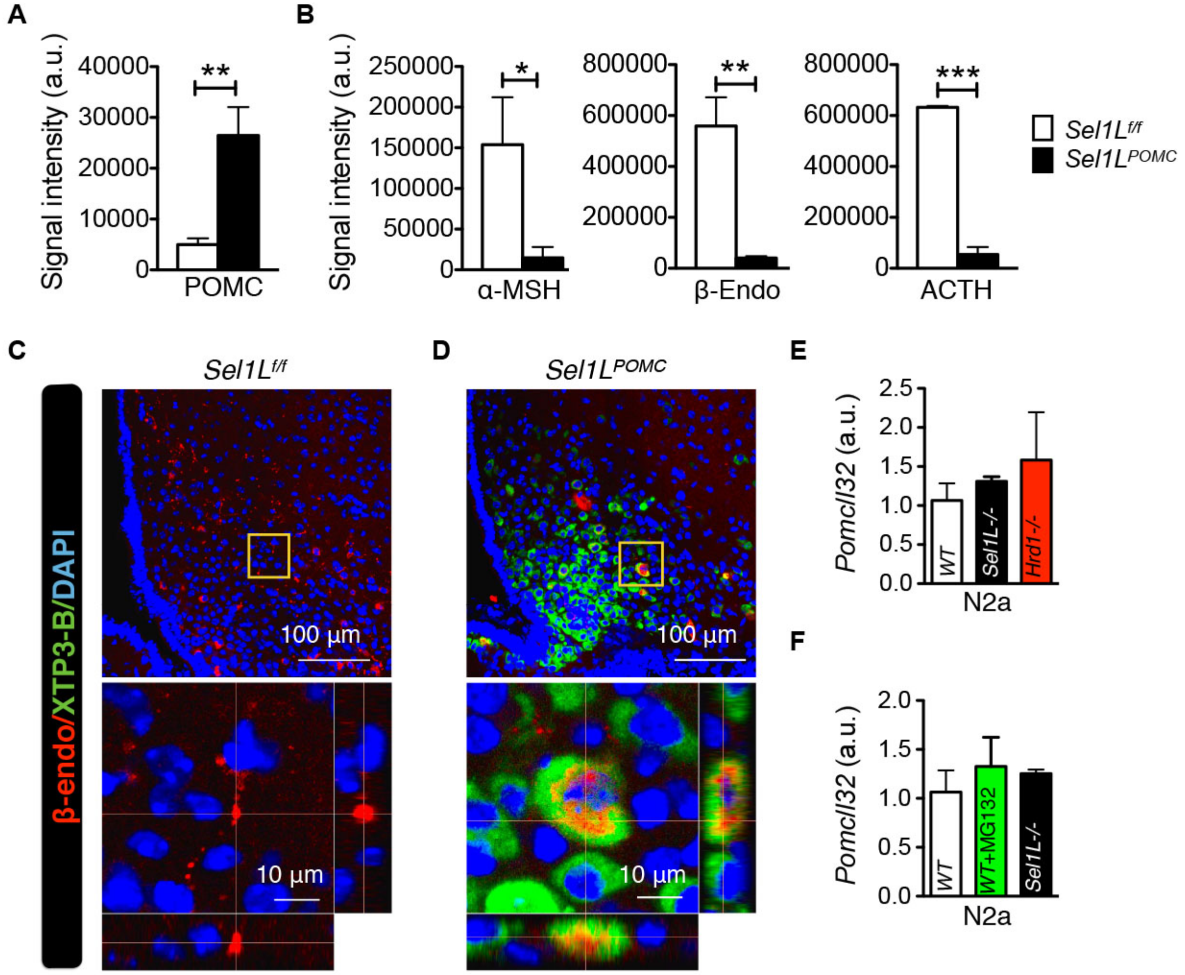

Figure S7. POMC C28F forms disulfide bond-mediated aggregates.
(A) POMC protein decay assay where POMC-Flag-expressing HEK293T cells were treated with the translation inhibitor cycloheximide (CHX) for the indicated time points. Quantitation of POMC protein decay is shown below. (B) Sequences of N-terminal POMC from different species, with two disulfide bonds and four cysteines highlighted. (C) Western blot analysis of POMC-*WT*, -*C28F*, -*C28S* and two double mutants (*C28F/C50S, C28S/C50S*) in transfected WT HEK293T cells under non-reducing (- β-ME) and reducing (+ β-ME) conditions. $(POMC)_2$ refers to POMC dimers. Representative data from at least two independent experiments shown.

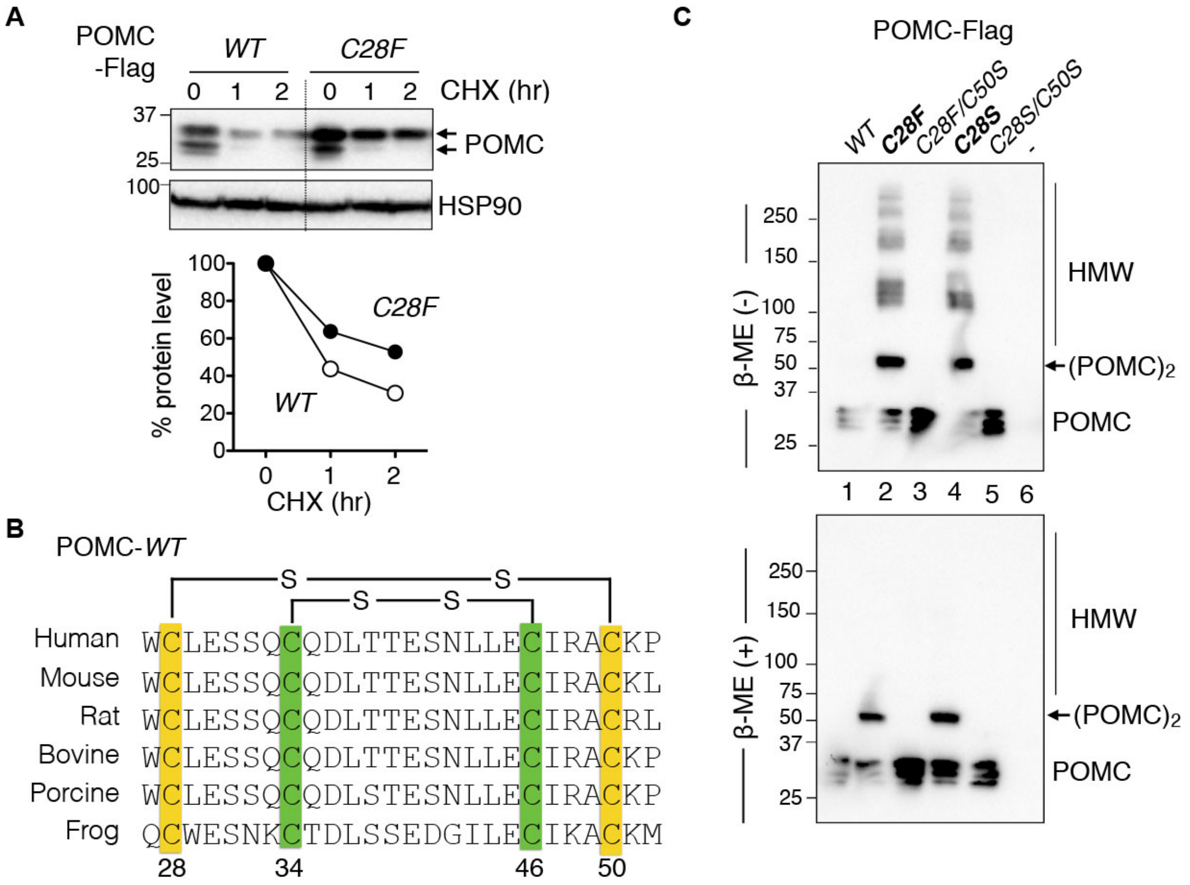

Figure S8. Aggregation of POMC C28F is mediated through unpaired cysteine residue 50.
(A) Western blot analysis of POMC in *HRD1-/-* HEK293T cells transfected with HRD1 (+) together with either POMC-C28F or -C28F/C50S, under non-reducing (-β-ME) and reducing (+β-ME) conditions. Note that POMC-C28F/C50S did not aggregate even in the absence of ERAD (Lane 3). (B) POMC protein decay assay where POMC-Flag-expressing HEK293T cells were treated with cycloheximide (CHX) for the indicated time points, with quantitation of protein decay shown on the below. Representative data from at least two independent experiments shown.

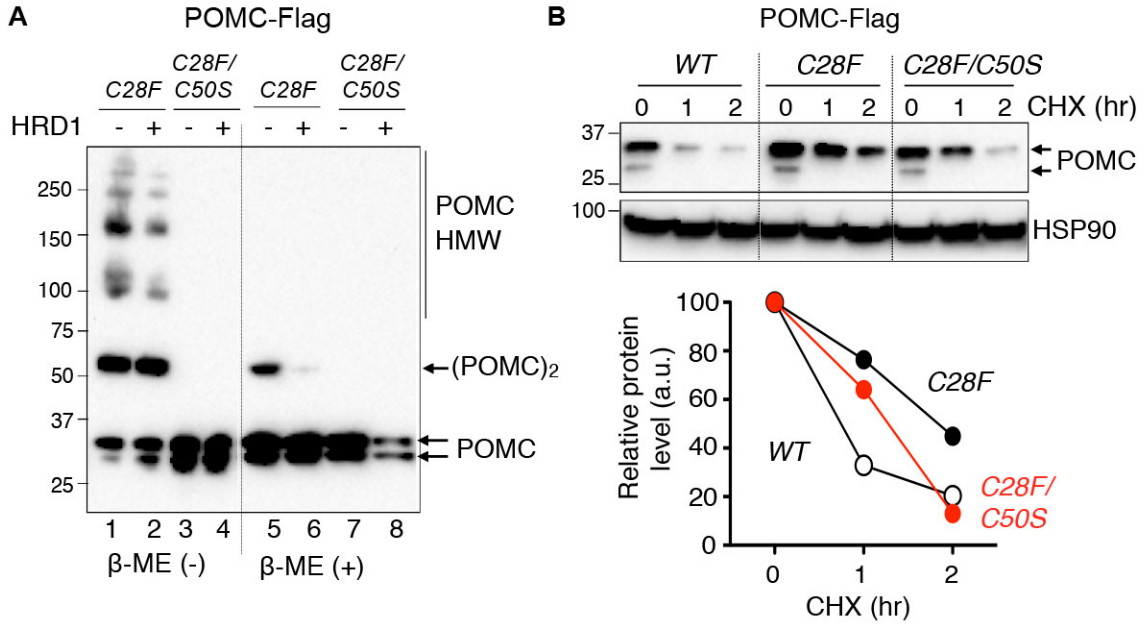

Materials and Methods

Metabolic cage studies. Metabolic cage analysis was performed with male mice at 8 weeks of age in the Comprehensive Lab Animal Monitoring System (CLAMS, Columbus Instruments) with 1 day acclimation followed by 2-day measurement. Animals were housed on a 12 hours light/dark cycle and controlled temperature (20 ~ 22 °C) in the Cornell University Weill Hall Mouse Facility.

Glucose (GTT) and insulin tolerance test (ITT). For GTT or ITT, male mice were fasted for 16 or 6 hours, respectively, followed by i.p. injection with glucose (2 g/kg body weight) or insulin (1 unit/kg body weight). Blood glucose was monitored using TRUEresult Glucometer (Nipro Diagnostics) at the indicated times.

Body composition. Mice were scanned using DEXA (PIXImus, GE Medical Systems Lunar) under anesthesia condition, according to manufacturer's instruction.

Multiplex assay. Serum levels of leptin and insulin of 8- and 40-week-old mice were determined by Bio-Plex Pro Mouse Diabetes 8-Plex Assay (171-F7001M, Bio-Rad, CA) and Bio-Plex ® MAGPIX™ multiplex Reader per manufacture's protocol.

Western blot. Frozen tissue was homogenized in lysis buffer [150mM NaCl, 50mM Tris pH 7.5, 10 mM EDTA, 1% Triton X-100 with freshly added protease (P8340, Sigma,) and phosphatase inhibitors (P5726, Sigma) and 1 mM DTT]. Tissue debris was discarded by centrifugation (13,000 g, 10 min at 4 °C). After the centrifugation, supernatant was collected, and protein concentration was measured using the Bradford assay (5000006, Bio-Rad). Protein was denatured by boiling in SDS loading buffer. Each sample was run with 15–30 µg total lysate. Primary antibodies were diluted in 5% milk/TBST or 2% BSA/TBST and incubated with PVDF membrane overnight at 4°C. Band density was quantitated using the Image Lab software on the ChemiDOC XRS+ system (Bio-Rad).

RNA isolation and quantitative-RCR. Brain tissues were microdissected and directly homogenized in Trizol reagent (15596-018, Invitrogen, CA). RNA was isolated per manufacturer's protocol. cDNA was synthesized with reverse transcriptase (18080-085, Invitrogen, CA). Gene expression was analyzed using ABI (QuantStudio 5). Primers were used for this study:
Pomc; 5'-AACGTTGCTGAGAACGAGTC-3', 5'-ACCTGCTCCAAGCCTAATG-3',
AgRP; 5'-GAATGGCCTCAAGAAGACAA-3', 5'-CGGTTCTGTGGATCTAGCAC-3',
Hrd1; 5'-AGCTACTTCAGTGAACCCCACT-3', 5'-CTCCTCTACAATGCCCACTGAC-3',
Sel1L;5'-TGGGTTTTCTCTCTCTCCTCTG-3', 5'-CCTTTGTTCCGGTTACTTCTTG-3',
Ire1α; 5'-ATCTGCGCAAATTCAGAACC-3', 5'-CTCCATGGCTTGGTAGGTGT-3',
Os9; 5'-GCTGGCTGACTGATGAGGAT-3', 5'-CGGTAGTTGCTCTCCAGCTC-3',
Bip; 5'-TGTGGTACCCACCAAGAAGTC-3', 5'-TTCAGCTGTCACTCGGAGAAT-3',
Ifng; 5'-GCGTCATTGAATCACACCTG-3', 5'-TGAGCTCATTGAATGCTTGG-3',
Il6; 5'-AGACAAAGCCAGAGTCCTTCAG-3', 5'-TGCCGAGTAGATCTCAAAGTGA-3',
Tnfα; 5'-TCAGCCGATTTGCTATCTCATA-3', 5'-AGTACTTGGGCAGATTGACCTC-3',
p65; 5'-GTGCCTACCCGAAACTCAAC-3', 5'-TGGGGGAAAACTCATCAAAG-3',
p50; 5'-TCAATGCTCAGGAGCAGAAG-3', 5'-GCCGCTATATGCAGAGGTGT-3',
Iκb; 5'-CTCCAGATGCTACCCGAGAG-3', 5'-CATTCTTTTTGCCACTTTCCA-3',
F4/80; 5'-TGCTGTTCAGAACCACAATACC-3', 5'- CACTGCCTCCACTAGCATCC-3',
Mcp-1; 5'- TTAAAAACCTGGATCGGAACCAA-3', 5'- GCATTAGCTTCAGATTTACGGGT-3',

TUNEL assay. Brain sections were mounted on gelatin-coated slides, dried at 55°C for 5 min and then permeabilized in 0.5% Triton X-100 at 85°C for 20 min. TUNEL assay was performed using In-Situ Cell Death detection kit (11684795910, Roche, Basel) per manufacturer's protocol. Brain section treated with DNase I (EN 0523, ThermoFisher Scientific, NH) for 30 min at 37°C were used as positive controls. Images were obtained by Nikon A1 Confocal Microscope at the Imaging Core of the University of Michigan Medical School.

H&E staining. Peripheral tissues were collected from mice fixed via transcardial perfusion with 4% PFA. Tissues were post-fixed in 4% PFA for overnight at 4 °C, washed with PBS and stored in 70% ethanol at 4 °C until dehydration for paraffin-embedding. Sectioning and H&E staining were performed on a fee-for-service basis by the Cornell/Michigan Histology Core Facilities.

Cell lines and transfection. HEK293T cells, N2a cells and AtT20 cells (all cell lines from ATCC) were cultured in DMEM (Corning, NY) with 1% Pen/Strip (Corning, NY) and heat inactivated 10% FBS (Hyclone), respectively. Cells were transfected within 24 hours after plating with PEI (HEK293T cells) or Lipofectamine 2000 (N2a cells).

Sucrose gradient sedimentation analysis. The assay was performed as described in (1). Briefly, transfected HEK293T cells were harvested and lysed in 1% NP40 lysis buffer as described in immunoprecipitation without any reducing reagents. Extracts were centrifuged through 3.7 ml of 5%– 25% sucrose gradients (prepared in 150 mM NaCl, 1 mM EDTA, 50 mM Tris HCl pH 7.5 and protease inhibitors) prepared freshly by progressively layering high- to low-density sucrose fractions in 5% increments in polyallomer tubes of 11×3×60 mm (Beckman Coulter, Brea, CA). Extracts were centrifuged at 48,000 rpm for 20 hours at 4°C using an SW 50 Ti rotor (Beckman Coulter). Each 4.2 ml gradient was divided evenly into 6 fractions (700 µl each). The pellet from fraction 7 was resuspended with 700 µl lysis buffer by pipetting and vortexing. Fractions were subsequently subjected to Western blot analyses under reducing and non-reducing conditions.

Drug treatment and non-reducing SDS-PAGE gel analysis. Preparation of cell and tissue lysates and western blots were performed as previously described (2). For cycloheximide (CHX) chase experiments, cells were treated with CHX treatment (10 µg/ml) at the indicated times. For denaturing SDS-PAGE, whole-cell lysates were first harvested in lysis buffer (150 mM NaCl, 1% Triton X-100, 1 mM EDTA, and 50 mM Tris HCl [pH 7.5]) supplemented with protease inhibitor cocktail (Sigma-Aldrich) and 1 mM DTT. Following incubation on ice for 25 min, lysates were centrifuged for 15 min. Supernatants were collected and denatured by the addition of 5X denaturing sample buffer (250 mM Tris HCl pH 6.8, 1% SDS, 50% glycerol, 1.44 M β-mercaptoethanol and 0.05% bromophenyl blue). Samples were boiled for 5 min and separated on a SDS-PAGE gel. For non-reducing SDS-PAGE gel analysis, cells were lysed in Triton X-100 lysis buffer (150 mM NaCl, 1 mM EDTA, 50 mM Tris pH 7.5, 1% Triton X-100) supplied with protease inhibitor (Sigma-Aldrich) and 10 mM N-ethylmaleimide (NEM) on ice for 20 min. After centrifugation, the supernatant was mixed with reducing or non-reducing sample buffer. The samples were then separated on SDS-PAGE for Western blot analysis. For reducing SDS-PAGE analysis, supernatants were mixed with 5X denaturaing sample buffer (250 mM Tris HCl pH 6.8, 1% SDS, 50% glycerol, 1.44 M β-mercaptoethanol and 0.05% bromophenyl blue), and incubate at 65ºC for 10 min prior to being separated on a SDS-PAGE gel.

Immunoprecipitation. Cells were lysed in IP lysis buffer (150 mM NaCl, 1 mM EDTA, 50 mM Tris-HCl pH 7.5, 1% NP-40, protease inhibitor and protein phosphatase inhibitor cocktails, and 10 mM NEM. A total of 2 mg protein lysate was incubated with antibody-conjugated agarose beads overnight at 4°C, with gentle rocking (anti-FLAG agarose beads, A2220, Sigma, MN). Immunocomplexes were washed (137 mM NaCl, 2 mM EDTA, 20 mM Tris-HCl pH 7.5, 10% glycerol) and eluted under denaturing or non-denaturing sample buffer as described below.

Pulse-chase analysis. Label of newly synthesized proteins with [^{35}S]-cysteine and methionine were performed as previously described (3).

References:

1. Shi, G., Somlo, D., Kim, G.H., Prescianotto-Baschong, C., Sun, S., Beuret, N., Long, Q., Rutishauser, J., Arvan, P., Spiess, M., and Qi, L. 2017. ER-associated degradation is required for vasopressin prohormone processing and systemic water homeostasis. J Clin Invest 127:3897-3912.
2. Sha, H., He, Y., Chen, H., Wang, C., Zenno, A., Shi, H., Yang, X., Zhang, X., and Qi, L. 2009. The IRE1alpha-XBP1 pathway of the unfolded protein response is required for adipogenesis. Cell Metab. 9:556-564.
3. Sun, S., Shi, G., Sha, H., Ji, Y., Han, X., Shu, X., Ma, H., Inoue, T., Gao, B., Kim, H., Bu, P., Guber, R.D., Shen, X., Lee, A.H., Iwawaki, T., Paton, A.W., Paton, J.C., Fang, D., Tsai, B., Yates Iii, J.R., et al. 2015. IRE1a is an endogenous substrate of endoplasmic-reticulum-associated degradation. Nat Cell Biol 17:1546-1555.

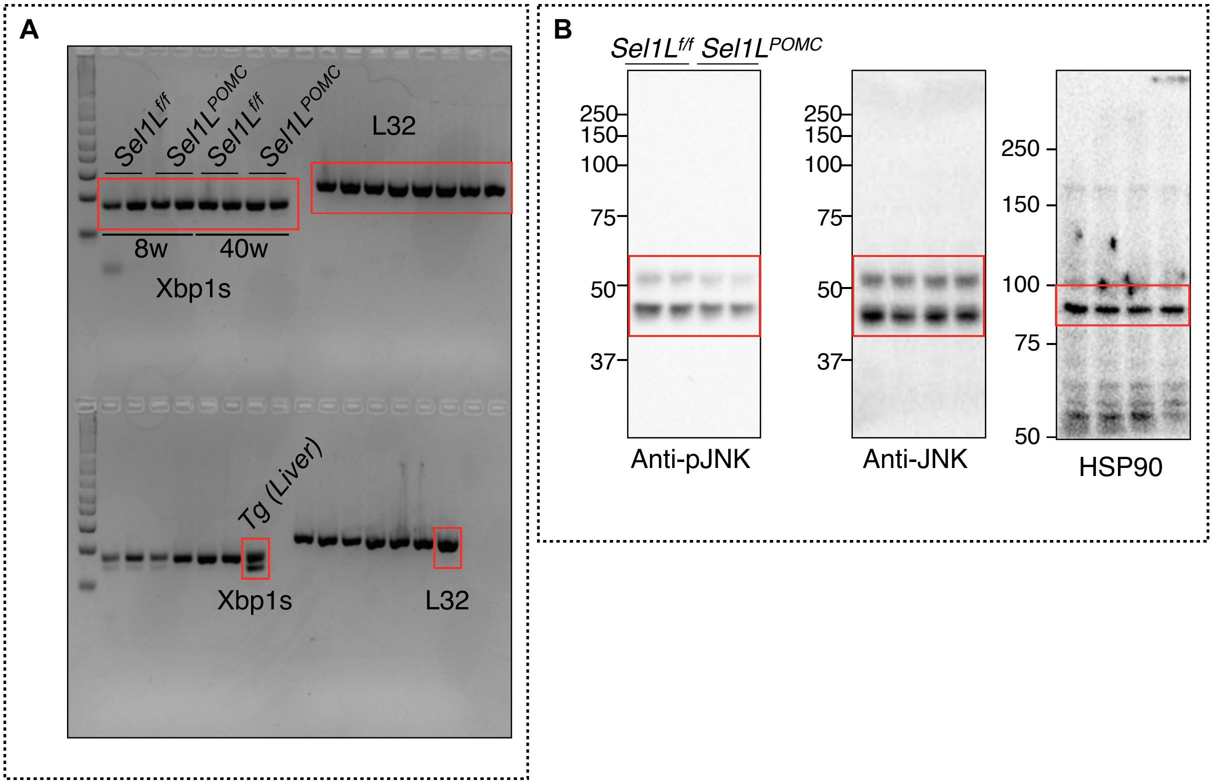

Full unedited gel for Figure 3

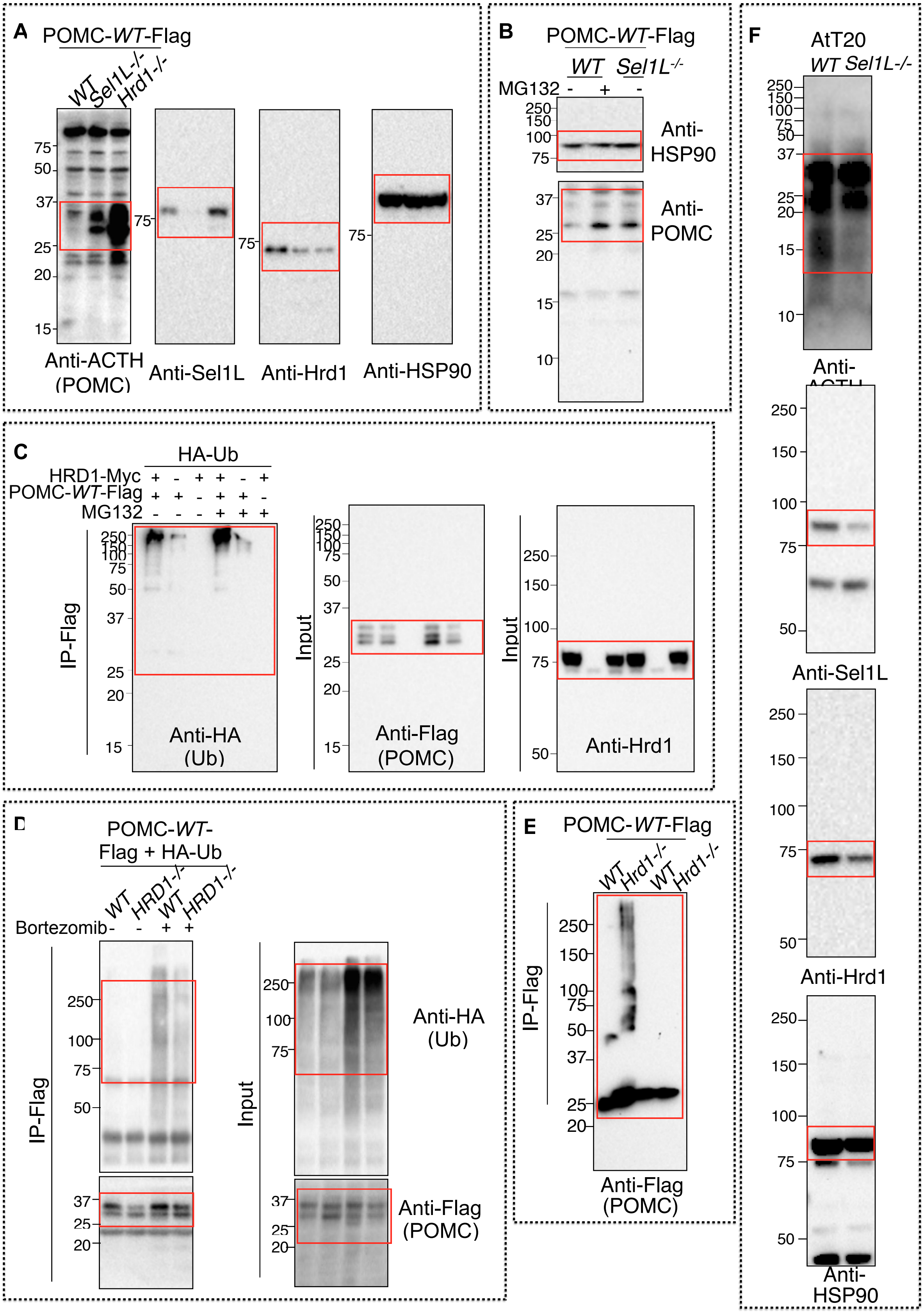

Full unedited gel for Figure 5

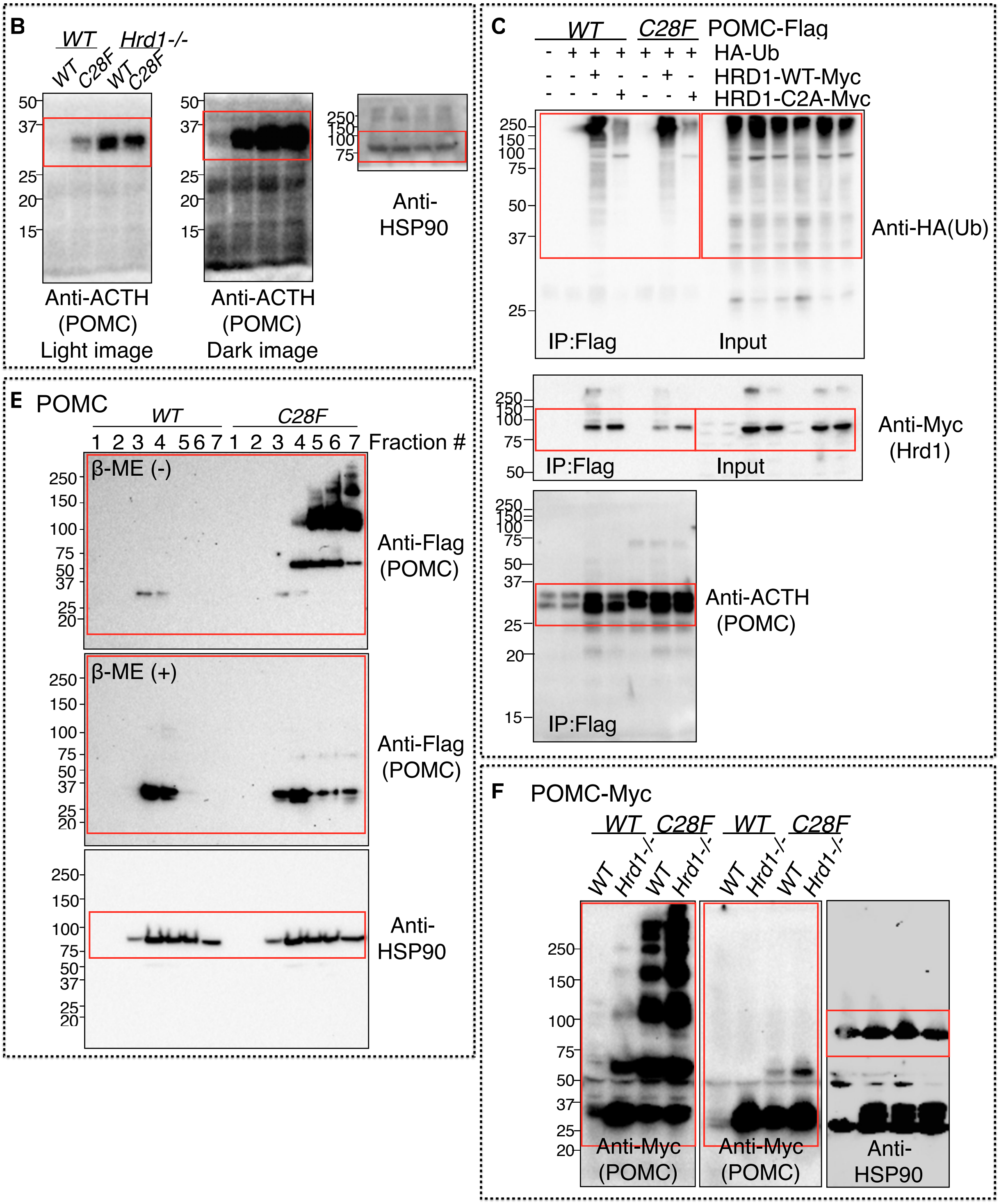

Full unedited gel for Figure 6

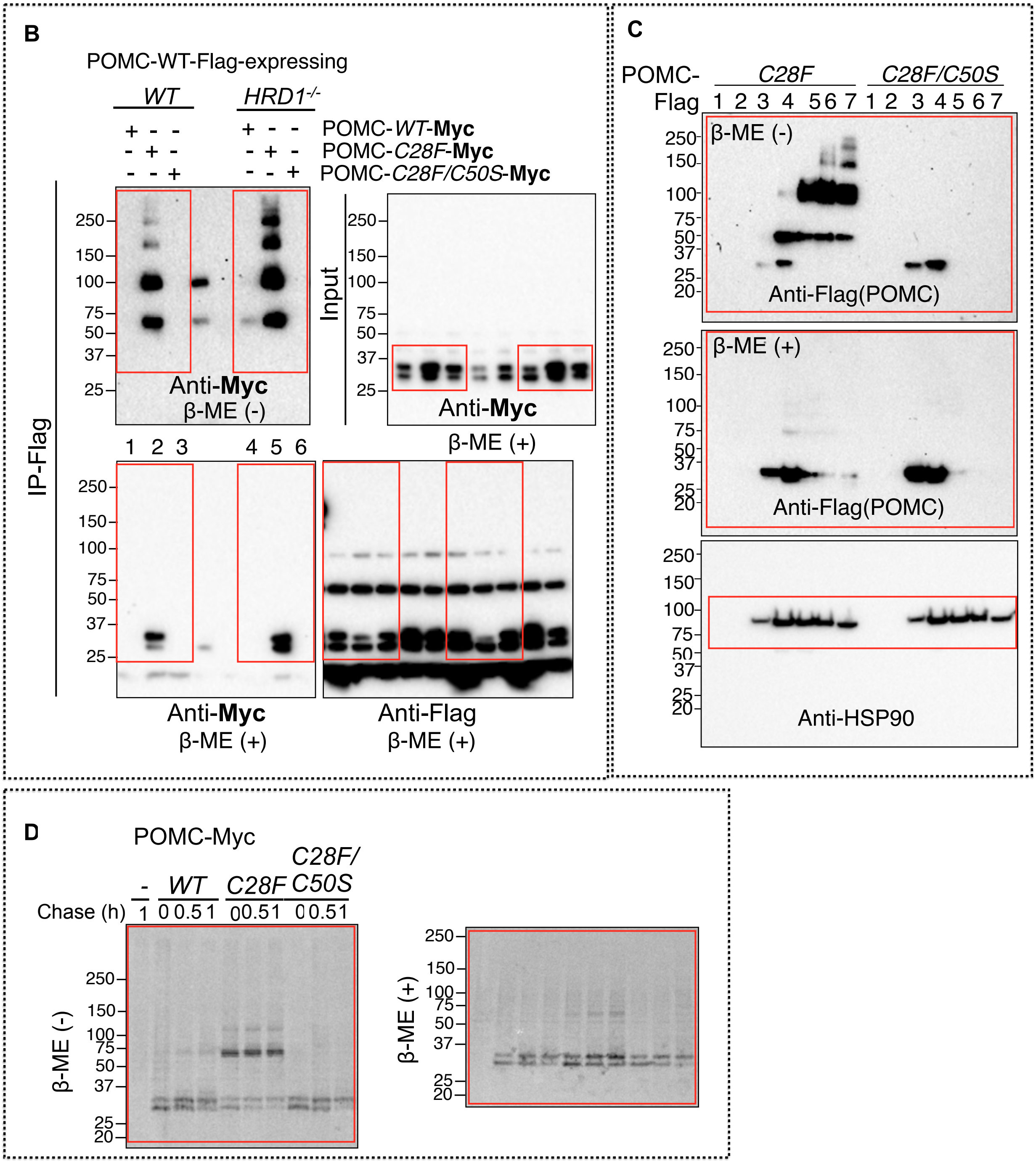

Full unedited gel for Figure 7

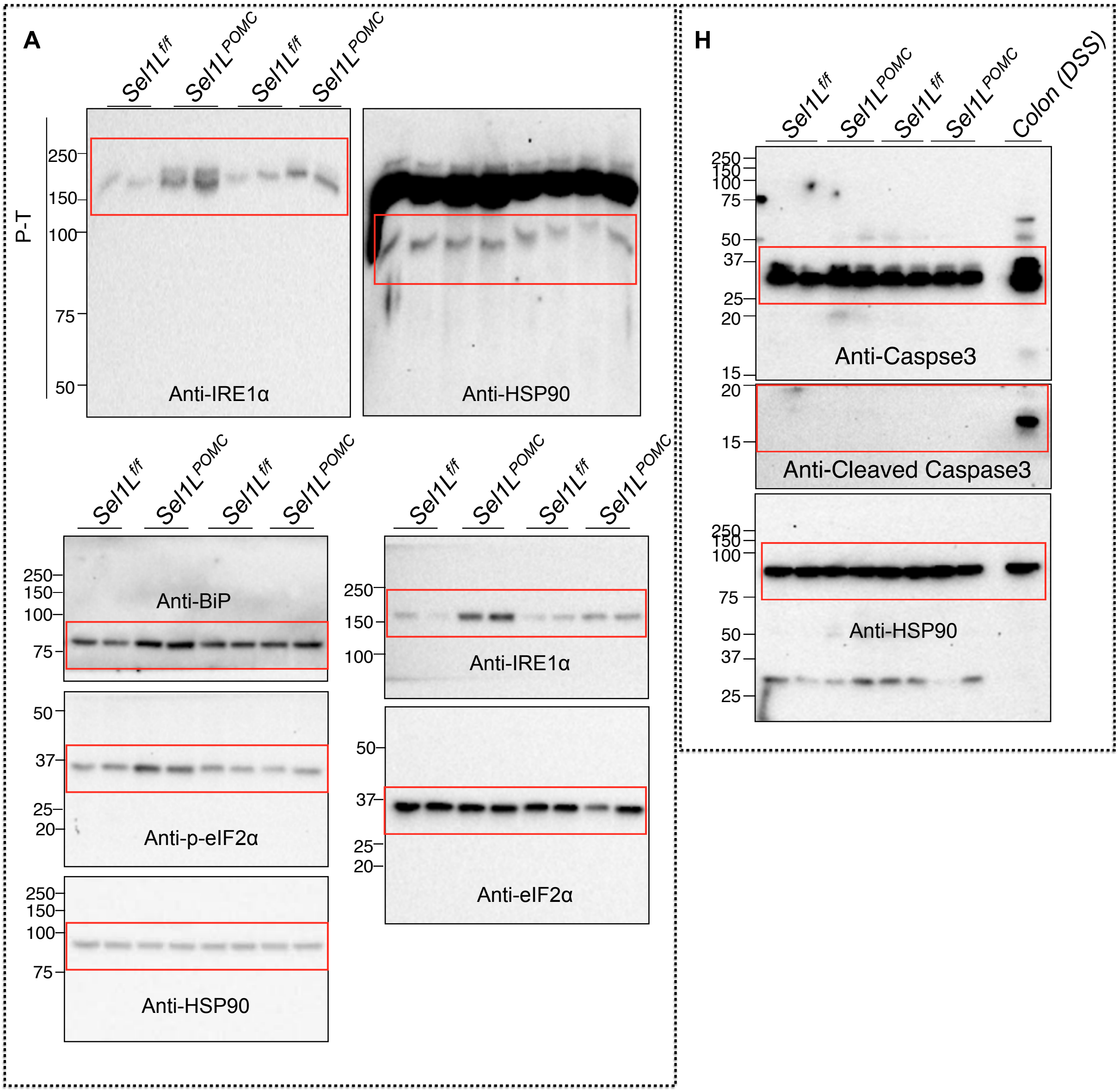

Full unedited gel for Supplementary Figure 5

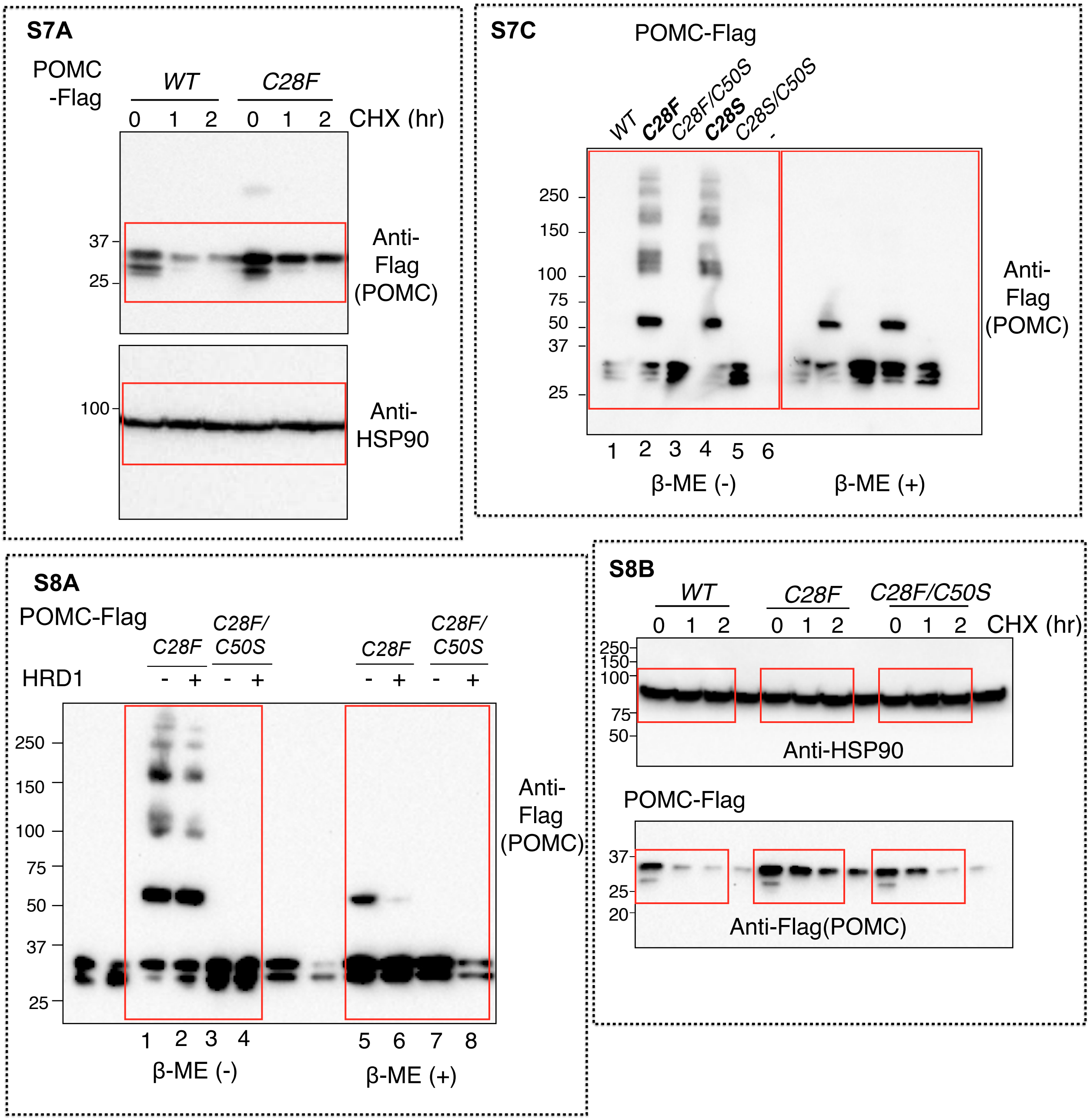

Full unedited gel for Supplementary Figure 7 and 8
